# Orco-dependent survival of odorant receptor neurons in ants

**DOI:** 10.1126/sciadv.adk9000

**Published:** 2024-06-07

**Authors:** Bogdan Sieriebriennikov, Kayli R. Sieber, Olena Kolumba, Jakub Mlejnek, Shadi Jafari, Hua Yan

**Affiliations:** ^1^ Department of Biology, New York University, New York, NY 10003, USA.; ^2^ Department of Biochemistry and Molecular Pharmacology, New York University School of Medicine, New York, NY 10016, USA.; ^3^ Department of Biology, University of Florida, Gainesville, FL 32611, USA.; ^4^ Center for Smell and Taste, University of Florida, Gainesville, FL 32610, USA.; ^5^ New York University Abu Dhabi, Abu Dhabi, United Arab Emirates.

## Abstract

Olfaction is essential for complex social behavior in insects. To discriminate complex social cues, ants evolved an expanded number of *odorant receptor* (*Or*) genes. Mutations in the obligate odorant co-receptor gene *orco* lead to the loss of ~80% of the antennal lobe glomeruli in the jumping ant *Harpegnathos saltator*. However, the cellular mechanism remains unclear. Here, we demonstrate massive apoptosis of odorant receptor neurons (ORNs) in the mid to late stages of pupal development, possibly due to ER stress in the absence of Orco. Further bulk and single-nucleus transcriptome analysis shows that, although most *orco*-expressing ORNs die in *orco* mutants, a small proportion of them survive: They express *ionotropic receptor* (*Ir*) genes that form IR complexes. In addition, we found that some *Or* genes are expressed in mechanosensory neurons and nonneuronal cells, possibly due to leaky regulation from nearby non-*Or* genes. Our findings provide a comprehensive overview of ORN development and *Or* expression in *H. saltator*.

## Teaser

Developing olfactory neurons in ants die after the onset of *odorant receptor* expression without the obligate co-receptor Orco.

## INTRODUCTION

**Fig. 1.**
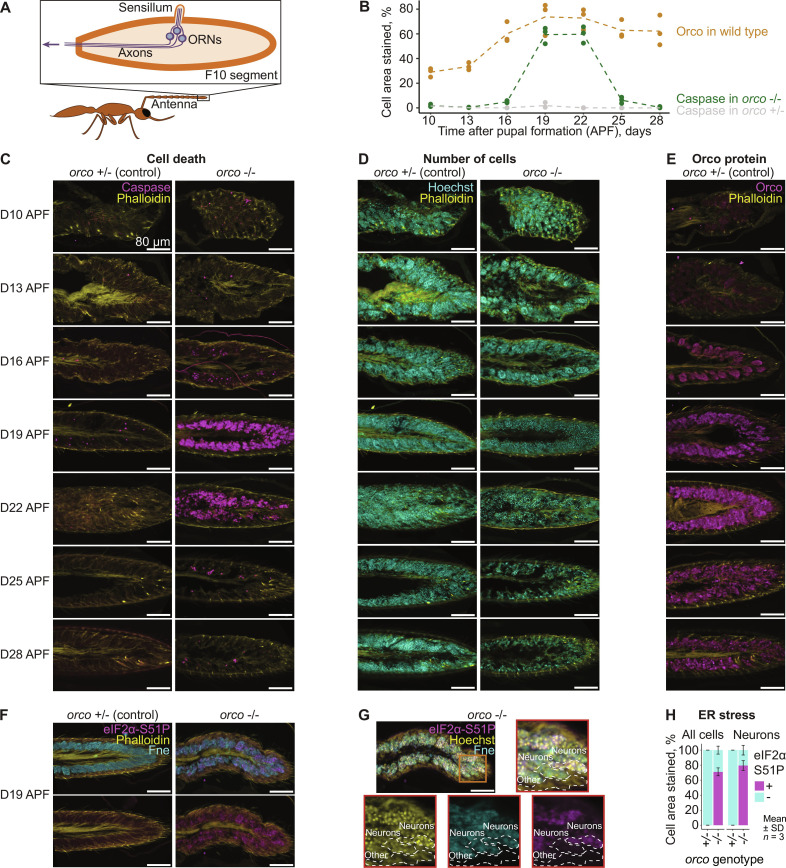
Temporal relationship between cell death and *orco* expression. (**A**) Simplified diagram illustrating the internal antennal structure of the olfactory system in ants. (**B**) Quantification of immunohistochemistry stainings shown in (C) to (E). Each individual point is a single replicate image. The dashed line connects the means of different time points for each protein-by-genotype combination. (**C** and **D**) Representative immunohistochemistry images of sectioned pupal antennae demonstrating the relationship between genotype [*orco* +/− (left) and *orco* −/− (right)], age [D10 APF (top) through D28 APF (bottom)], and cell death. Cell death is shown by the apoptotic marker cleaved caspase-3 (magenta) in (C), and temporal changes in cell density are shown via Hoechst (cyan) in (D). (**E**) Representative immunohistochemistry images of sectioned pupal antennae demonstrating the relationship between age [indicated as in (C) and (D)] and prevalence of Orco (magenta). (**F** and **G**) Representative immunohistochemistry images of sectioned pupal antennae demonstrating the relationship between genotype [*orco* +/− (left) and *orco* −/− (right)] and ER stress in the stage of peak apoptosis (D19 APF). ER stress is marked by phospho-eIF2α (Ser^51^) (magenta), and neurons are marked by Fne (cyan). The rightmost panels include a nuclei marker (Hoechst) and a magnified view of the distal end of the flagellum (red box), where three distinct groups of cells can be seen: (i) neurons experiencing ER stress (top dashed cluster), (ii) neurons not experiencing ER stress (middle dashed cluster), and (iii) nonneuronal cells not experiencing ER stress (bottom dashed cluster). (**H**) Quantification of immunohistochemistry staining shown in (G). Stacked bar plot represents average percent of cell or neuron area stained for phospho-eIF2α (Ser^51^) across replicates. Magenta, phospho-eIF2α (Ser^51^)–positive area; cyan, phospho-eIF2α (Ser^51^)–negative area.

The emergence of eusociality is a major evolutionary transition (*1*). Eusociality refers to a complex social system in which overlapping generations live together, wherein one or a few colony members reproduce and others cooperate for brood care or other specialized tasks, known as division of labor (*2*). This system is observed in several insect families, including ants, bees, and wasps in the order Hymenoptera, as well as termites, which have all evolved highly cooperative social behavior. In these insects, social recognition is critical for maintaining proper colony function. Sensory systems, and chemosensory communication via pheromones in particular, play a crucial role in social behavior by allowing individuals to recognize and communicate with each other. Antennae are the major anatomical organs involved in insect chemosensation. They have hair-like structures called sensilla, which house the dendrites of odorant receptor neurons (ORNs) (Fig. 1A). The pheromone-sensing role of ORNs is essential for eusocial insects to achieve social recognition. Thus, deciphering the mechanisms underlying social communication requires understanding the development and function of ORNs.

The architecture of the olfactory system in insects (predominantly studied in *Drosophila*) largely follows the rule of “one neuron, one receptor,” similar to vertebrates (*3*). Each ORN selectively expresses a single odor-sensing (tuning) receptor from one of the three major gene families—*odorant receptors* (*Or*), *ionotropic receptors* (*Ir*), or *gustatory receptors* (*Gr*) (*4*, *5*). While all vertebrate and nematode olfactory receptors are G protein–coupled receptors (*6*, *7*), tuning ORs in insects bind to an obligate co-receptor (Orco), forming ligand-gated ion channels (*8*–*12*). Most insect species, including *Drosophila*, have fewer than 100 *Or* genes (*4*, *13*); however, this number is markedly expanded in hymenopteran insects, a feature considered pre-adaptive to social evolution (*5*). Further expansion in ants (300 to 500 *Or* genes) may have facilitated the recognition of complex social cues. In *Drosophila*, all ORNs that express the same tuning *Or* project axons to the same glomerulus in the antennal lobe (AL) of the brain, where they synapse onto projection neurons that transmit information into the central brain (*3*, *5*). Consistent with the expansion of *Or* genes in ants, the number of AL glomeruli has also increased from 60 in *Drosophila* to 270 to 500 in female ants (*14*–*16*). In summary, ants have an expanded number of both *Or* genes and AL glomeruli, but it is unclear whether the same developmental paradigms as in *Drosophila* exist in ants to control the production of this expanded array of ORNs.

Until recently, studies of the biological function of ORs and ORNs in eusocial insects were limited by a lack of genetic tools. This has been overcome by the development of *orco* mutant ants via CRISPR/Cas9 and subsequent phenotypic analyses (*15*, *16*). We have previously found that *orco* mutation in the jumping ant *Harpegnathos saltator* abolishes all OR-mediated olfactory sensation. Furthermore, mutant animals display communication deficits and abnormal behaviors, such as “wandering” outside the nest, antennal twitching in the absence of an external stimulus, and inability to communicate with conspecifics (*16*). Unlike in *Drosophila* where Orco is only required for the prolonged survival of ORNs in adults but not for their development (*17*, *18*), the loss of Orco in *H. saltator* markedly reduces the number of ORNs in antennae and the number of AL glomeruli in newborn ants (*16*). The same phenotypes have been found in the clonal raider ant *Ooceraea biroi* (*15*) and the honeybee *Apis mellifera* (*19*), showing that social hymenopterans with their expanded *Or* repertoire require Orco for proper ORN development.

The exact sequence of developmental events and the underlying molecular mechanisms leading to the reduced numbers of antennal cells and AL glomeruli observed in *orco* mutants remain unknown. Are these phenotypes a result of ORN apoptosis or other developmental events? If the former, is it due to improper receptor trafficking or to the absence of neuronal activity (*20*, *21*)? We show that all *Or*-expressing ORNs undergo massive apoptosis when Orco is absent, possibly triggered by endoplasmic reticulum (ER) stress. Only a small proportion of *orco*-expressing cells survive. These ORNs coexpress genes encoding IRs along with IR co-receptors (Ircos). In addition, we found that some *Or* genes are also expressed in nonneuronal antennal cells that do not need Orco to survive. This *Or* gene expression might be spurious due to the leaky regulation of a neighboring gene in these cells.

## RESULTS

### ORNs undergo apoptosis during pupal development in *orco* mutants

To address the events leading to the reduction in antennal ORNs in adult *orco* mutant ants, we performed immunostaining of antennae across pupal development. The duration of pupation in *H. saltator* is approximately 30 days (*22*). Homozygous *orco* mutant and heterozygous control (*16*) pupae were harvested at seven developmental stages: D10, D13, D16, D19, D22, D25, and D28, representing 10 to 28 days after puparium formation (APF). Their antennae were immunostained with antibodies against cleaved caspase-3 as a marker of programmed cell death (apoptosis), in conjunction with the nuclear marker Hoechst to confirm the loss of ORNs in late-stage pupae. In *Drosophila*, apoptosis of some ORNs normally occurs during neurogenesis (22 to 24 hours APF, which roughly translates to D7 APF in *H. saltator*) to eliminate extraneous ORNs during asymmetrical divisions of sensory organ precursor (SOP) cells (*5*, *23*–*26*). Consistently, in heterozygous ant pupae, we observed occasional apoptosis (~0 to 2% of total cells in the antenna) at the earliest time point examined (D10 APF), and a second small increase in the number of apoptotic cells at D19 (Fig. 1, B and C), although the cause remains unclear. In the early days of development, homozygous pupae resembled heterozygotes closely in patterns of cell death, only beginning to diverge around D16. In *orco* mutant pupae, apoptosis greatly increased and was much more prevalent than their heterozygous counterparts, peaking between D19 and D22, and then ceasing around D25 to D28 (Fig. 1, B to D). Within the antennae, neuronal cells can be differentiated from other cell types based on location (deep below the cuticle, around the central axon bundle) and the shape of their nuclei (round and in cell clusters). Furthermore, neuronal cells (especially ORNs) make up the majority of antennal cells. On the basis of these features, we could infer that the dying cells are ORNs. In summary, a massive wave of apoptosis in mid-pupation (D19 and D22) leads to a greatly reduced ORN population by the end of pupation.

We also tracked the temporal pattern of Orco protein expression in developing heterozygous pupae. In pupae younger than D16, Orco stained faintly (Fig. 1, B and E). Then, the intensity of Orco staining greatly increased and peaked at D19 (Fig. 1, B and E). Orco stained cells in grape-like clusters from D10 to D16 (Fig. 1E). Beyond this period, the cell clusters became larger, while the boundary between cells was less obvious (Fig. 1, D and E). Therefore, the massive apoptosis in homozygous mutant pupae (D19 to D22) occurs after a marked increase in Orco protein in the control pupae. Homozygous pupae were also stained in parallel as a control for our Orco antibody; as expected, their antennae did not exhibit any Orco signal (fig. S1).

A potential cause of ORN apoptosis is disrupted trafficking of OR proteins to dendrites. In *Drosophila*, Orco plays a role in protein trafficking (*27*), the disruption of which could result in accumulation of OR proteins in the ER. This results in cellular stress and eventually apoptosis. The role of Orco in ER stress has not been established in ants. To explore this possibility, we performed further immunostaining to target eIF2α phosphorylated at Ser^51^, a marker of ER stress in *Drosophila* (*28*). We stained D19 APF antennae of heterozygous and homozygous individuals, as this stage correlates with peak apoptosis. Costaining with a neuronal marker (Fne, LOC105190174) showed that many (but not all) neurons in homozygous pupae experience ER stress (Fig. 1, F to H); however, due to the antibodies for phospho-eIF2α and cleaved caspase-3 sharing the same host species, we could not confirm that it was specifically the stressed cells that were undergoing apoptosis. Additionally, we looked for evidence of expression changes of ER stress marker genes in RNA-sequencing (RNA-seq) data (see below) but found little difference between mutant and wild type (WT) (fig. S2, E and F). In summary, the massive wave of apoptosis in the mutant mid-pupae may be triggered by ER stress, as suggested by eIF2α-Ser51P staining. The ER stress may be caused by the inability of OR to traffic to dendrites without Orco.

### ORNs express *Or* genes before cell death in *orco* mutants

After establishing the potential link between ORN apoptosis and the increase in Orco expression, we asked two questions aiming to further understand the developmental context of these events: (i) Does the cell death promptly follow neurogenesis or does it occur at a later developmental stage? (ii) Are tuning *Ors*, like *orco*, expressed before the initiation of cell death? To address these questions, we performed bulk RNA-seq on WT and mutant antennae at D10, D15, D20, and D25 APF.

**Fig. 2.**
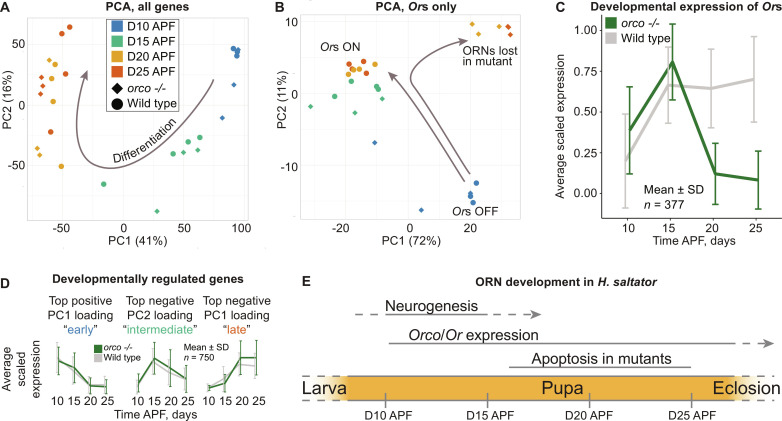
Developmental trajectory in WT and *orco* mutant antennae. (**A**) Whole transcriptome–based PCA of bulk RNA-seq samples collected from WT and mutant pupae of different ages. Percentages represent the amount of variance explained by each PC. The arrow illustrates the developmental trajectory from D10 to D25. (**B**) PCA based on *Or* genes only. The arrows illustrate WT and mutant developmental trajectories. (**C**) Expression pattern of *Or* genes. The line represents average expression across genes after scaling the expression of each gene to its maximum expression value. The error bars represent SD in the scaled expression values across genes. (**D**) Identification strategy and expression patterns of the “early,” “intermediate,” and “late” gene sets. The line represents average expression across genes after scaling the expression of each gene to its maximum expression value. The error bars represent SD in the scaled expression values across genes. (**E**) Summary of olfactory developmental process in *H. saltator*: Cell divisions that presumably produce neurons occur during D10 to D15 (APF); *Or* and *orco* mRNAs are visible at D10 and reach their peaks at D15, and Orco protein level increases from D10 and peaks at D19; in the mutant, apoptosis starts at D16, peaks at D19 to D22, and fades at D25.

Principal components analysis (PCA) showed that developmental stage was the primary source of variance in gene expression (Fig. 2A). We identified that “early genes” (expression peaking at D10) were enriched for mitotic terms, “intermediate genes” (expression peaking at D15) were enriched for terms associated with differentiation of neurons and nonneurons, as well as neuronal activity, and “late genes” (expression plateauing at D20) were enriched for metabolic terms (Fig. 2D and fig. S2, A to C and G). These data suggested that the peak of cell death in *orco* mutants observed at D19 and D22 occurred long after SOP division (unlike the programmed cell death in dividing ORNs in *Drosophila*).

For *Or* genes, both PCA (Fig. 2B) and developmental expression profile of *Or* genes (Fig. 2C) showed that expression of *Ors* started at D10, increased markedly between D10 and D15, and remained largely stable after D15 in WT pupae. In *orco* mutant pupae, however, although a similar pattern was observed at D10 and D15, *Or* expression dropped sharply between D15 and D20 and remained low at D25 (Fig. 2C). A parallel pattern was observed with *orco*: its mRNA level dropped sharply after D15 in the mutants (fig. S2D). These data allowed us to infer the following developmental sequence: Cell proliferation occurs around D10, followed by ORN differentiation at D15 to D16 with peak expression of *orco* and *Or*s. Concurrently, apoptosis commences in the *orco* mutants and then intensifies markedly, peaking at D19 to D22 (Fig. 2E). The death of cells leads to a sharp decline in the expression of tuning *Or*s in the mutants. In summary, massive apoptosis occurs in differentiating neurons that express *Ors* and *orco*.

### Functional *Ir* complex genes are coexpressed in surviving *orco*-expressing ORNs

**Fig. 3.**
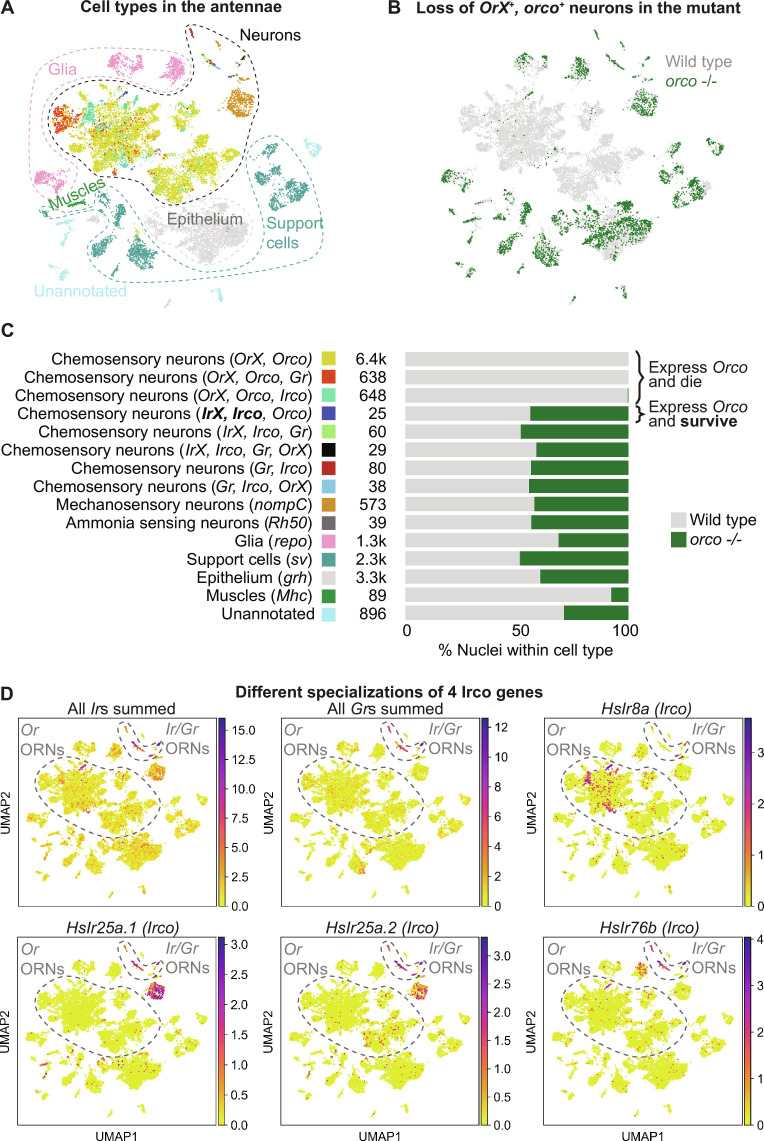
Cell death at single-cell resolution. (**A**) Combined UMAP plot showing the distribution of different cell type categories from (C). (**B**) Same UMAP plot as in (A), but with color showing WT and mutant cells. (**C**) List of cell type categories used in this study. Category labels are based on expression data only and should not be interpreted as functional descriptions because some neurons may be polymodal, and molecules traditionally considered chemoreceptors may have functions beyond the reception of chemical signals. Colored rectangle near each category shows which color was used for nuclei of this category in (A). The number is the total number of nuclei from the category in the combined dataset. The stacked bar plot shows the proportion of WT and mutant nuclei from the category in the combined dataset. (**D**) Combined expression of all *Ir*s, all *Gr*s, and the expression of the four *irco* genes plotted on the UMAP.

To gain insight into what cell types undergo apoptosis in *orco* mutants, we performed single-nucleus RNA-seq (snRNA-seq) of WT and mutant antennae. Our dataset contained ~16,000 nuclei, including ~13,000 WT nuclei and ~3500 nuclei from the mutant (Fig. 3, A and B). We first observed that neurons made up strikingly different proportions of the total number of nuclei in WT (67%) and mutant samples (12%). Such marked underrepresentation of neurons in mutants indicated that neurons are disproportionately affected by cell death, consistent with our caspase staining. Thus, we set out to investigate the specific types of neurons that were absent in the mutants (Fig. 3C). We classified individual neuronal cells based on the repertoire of receptors they expressed, specifically *Gr*s, tuning *Ir*s, *Ir* co-receptors (*irco*s), tuning *Or*s, *orco*, the ammonia transporter *Rh50*, and the mechanoreceptor *nompC* (see Materials and Methods for details). This allowed us to place the neurons into categories as shown in Fig. 3C, e.g., “mechanosensory neurons” or “chemosensory neurons expressing *Gr*(s) or *Ir* and their co-receptor(s).” Notably, we observed several cell types coexpressing *irco*s with *orco* and tuning *Or*s. In *Drosophila*, the *irco* gene *Ir25a* is expressed in most *Or/Orco*-positive ORNs. In contrast, the two ant orthologs of *irco Ir25a* (*HsIr25a.1* and *HsIr25a.2*) were only expressed in *Ir*-, *Gr*-, and *nompC*-positive neurons. In contrast, another *irco*—*HsIr8a*—was coexpressed with some *Orco/Or*-positive ORNs (Fig. 3D). The fourth *irco* gene, *HsIr76b*, displayed a similar expression pattern to that of *HsIr25a.1* and *HsIr25a.2*, with the exception of a peculiar cluster of *orco*-positive cells that coexpressed it with *orco*, *HsIr8a*, and a tuning *Ir* (see below).

There were almost no mutant nuclei belonging to the *Or/orco*-expressing ORNs (*OrX*, *orco*) (Fig. 3, B and C), indicating that Orco is necessary for the survival of the ORNs that express tuning *Ors*. Neurons that expressed *Gr*(s) or *irco*(s) in addition to a tuning *Or* and *orco* were, likewise, almost completely absent from the mutant sample. Thus, the additional expression of these receptors or co-receptors was not sufficient to rescue the effect of the *orco* mutation. However, neurons that expressed both tuning *Ir*(s) and *irco*(s) in addition to *orco* alone fully survived in the mutants (Fig. 3, B and C). This showed that Orco is not necessary for the survival of ORNs that express tuning *Ir*s and *Irco*s. Finally, neurons that did not express *orco* and nonneuronal cells were not affected in the mutants, with a surprising but currently unexplained exception of muscle cells, which were reduced in the absence of Orco (Fig. 3C). In summary, we observed that the antennae of *orco* mutants specifically lacked ORNs expressing tuning *Or*s and *orco*, including those cells that additionally expressed *Gr*s or *irco*s without tuning *Ir*s. In contrast, some ORNs expressing *orco* were maintained in the mutants as long as they additionally produced a functional IR/Irco complex.

### Nonneuronal cells expressing *Or*s without *orco* remained in *orco* mutants

**Fig. 4.**
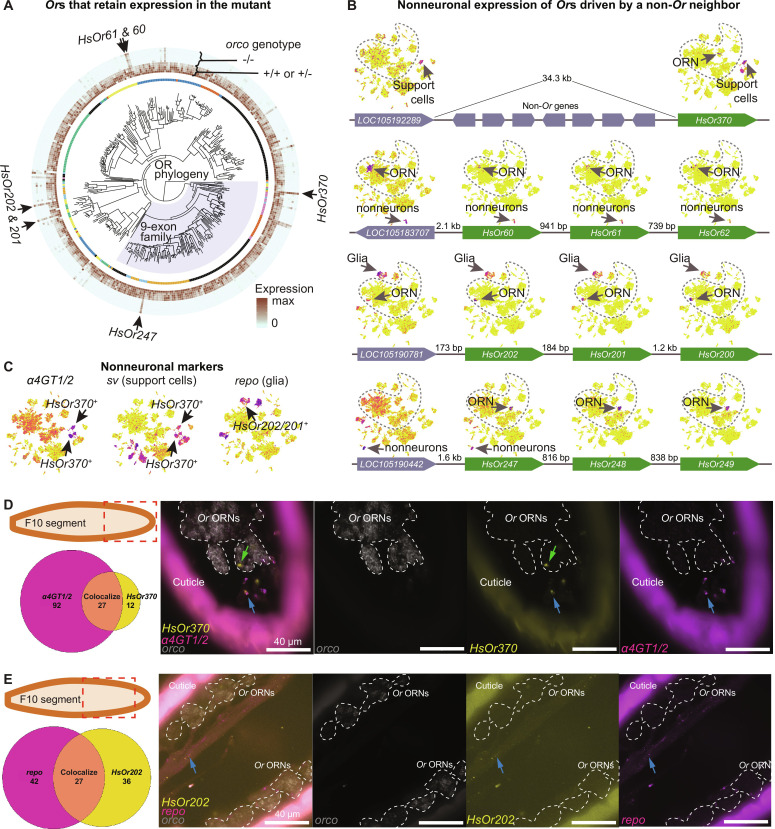
Identification of *Or*s that retain their expression in the mutant and their nonneuronal expression. (**A**) In the middle, the phylogeny of *Or* genes with the pheromone-sensing nine-exon *Or*s shaded blue. The inner circle depicts the genomic location of each gene such that genes on the same scaffold are colored identically. The outer circles show scaled expression in WT/heterozygote and mutant samples. Arrows point at genes whose expression is most prominently maintained in the homozygous mutant. (**B**) UMAP plots showing the expression of the *Or* genes that are marked with arrows in (A) and their neighbors. Dashed line encircles neurons. Green boxes correspond to *Or* genes, and gray boxes correspond to non*-Or* genes. The arrow at the end of each box denotes the 3′ end. (**C**) UMAP plots showing the expression of selected tissue markers. (**D**) HCR RNA-FISH showing overlap between *HsOr370* (yellow), identified support cell marker α*4GT1/2* (magenta), and a neuronal marker (*orco*, gray). Green arrows show cases of *HsOr370* expression in neuronal cells. Blue arrows demonstrate cases where *HsOr370* colocalizes with support cell markers, but not the neuronal marker. The red box in the diagram illustrates the region of the flagellum (as illustrated in Fig. 1A) imaged. The Venn diagram shows the total counts of cells coexpressing *HsOr370* and α*4GT1/2* and cells expressing either gene alone in five sampled images. (**E**) Same as (D), but for *HsOr202* (yellow) and the glial marker *repo* (magenta).

We also performed bulk RNA-seq on the antennae of adult WT, heterozygous, and homozygous *orco* mutants. Having established that most *Or*-expressing ORNs die, we expected to see a drastic reduction in *Or* expression in the mutant antennae. This was the case for most *Or*s in the homozygous mutants (Fig. 4A). Unexpectedly, several *Ors* not closely related phylogenetically, such as *HsOr370*, *HsOr61*, *HsOr247*, and *HsOr202*, retained considerable expression in the mutants (Fig. 4A). We sought to confirm this observation using our snRNA-seq data (fig. S4A). Plotting the expression of these genes on the Uniform Manifold Approximation and Projection (UMAP) revealed that these *Or* genes were expressed both in neurons and in various populations of nonneuronal cells including specific subtypes of glia and support cells (Fig. 4, B and C, and fig. S4C). Thus, only their ORNs died, while non-ORN cells survived in *orco* mutants, which explained why they remained detectable. In mammals, some *Or* genes exhibit expression outside olfactory cells, and *Or* genes with such expression patterns are located near non-*Or* genes expressed in the same non-olfactory tissues (*29*–*31*). Accordingly, we found that non-*Or* genes neighboring *HsOr370*, *HsOr61*, *HsOr247*, and *HsOr202* exhibited expression in the same nonneuronal tissues as these *Or*s (Fig. 4B). In some clustered *Or*s, we noted a pronounced inverse relationship between the strength of the nonneuronal expression and the physical distance to the non-*Or* gene: *HsOr202*, which is the closest to the glia-expressed non-*Or* gene *LOC105190781*, had the strongest expression in glia, while more distant *HsOr201* and *HsOr200* had weaker expression in glia (Fig. 4B and fig. S4C). Additionally, *Or* expression in nonneuronal tissues tended to be lower than in ORNs (fig. S4B), consistent with the idea that this “ectopic” expression is driven by the regulatory elements of neighboring genes. We proceeded to systematically analyze *Or* expression in the mutant and found many additional cases of expression in nonneuronal tissues as well as several cases where *Or* genes were expressed in mechanosensory neurons, which also survived in the mutant (table S3). Of note, none of these non-ORN cell types expressed *orco*. In summary, the *orco* mutants lost the expression of most *Or* genes while retaining the expression of some *Ors* in certain nonneuronal cells (such as support cells and glia) or non-olfactory neuronal cells (mechanosensory neurons). This expression is independent of *orco* and may be driven by closely located regulatory elements of non-*Or* genes, leading to leaky expression of the *Or* genes (*32*, *33*). A majority (~70%) of *Or* expression in the non-ORNs can be explained by the leaky regulation of neighboring genes, while ~30% of *Or* expression cannot (table S3) (see Discussion).

To explore the localization of *Or*s in adult antennae, hybridization chain reaction (HCR) RNA fluorescence in situ hybridization (RNA-FISH) probes were designed for *HsOr206* (an *Or* gene solely expressed in ORNs) and *HsOr370* (an *Or* that is expressed in nonneuronal cells in addition to ORNs). *HsOr206* was always expressed with neuronal markers (i.e., *Syt1* and *orco*); however, *HsOr370* was also expressed in apparently nonneuronal cells, which exhibited an elongated structure (fig. S4D). Our snRNA-seq data showed that *HsOr370* was expressed in two clusters of *sv*-expressing support cells (Fig. 4, B and C). We selected marker genes and designed probes for these clusters. Two of the selected genes, α*4GT1/2 (LOC105190591)* and *LOC105182650*, were expressed in both *HsOr370*-positive clusters, while *LOC105188530* was only expressed in one of them (Fig. 4C and fig. S4E). Cells expressing these genes generally appeared just under the cuticle. *HsOr370* was also expressed in *orco*-expressing ORNs, thus exhibiting expression in both neuronal and nonneuronal cells (Fig. 4D and fig. S4F). Unlike our other support cell markers, *LOC105182650* stained cytoplasm. The unusual tubular shapes of these cells further confirmed their cell type (fig. S4G). Furthermore, the cytoplasm of these cells seemed to project through the pores of the cuticle toward the sensilla. The localization and shape of our identified nonneuronal cells suggested that they could play a role in supporting the function of ORNs (*34*). In addition, HCR RNA-FISH allowed us to confirm the expression of *HsOr202* in glial cells (Fig. 4, B and C) that were present around antennal ORN axons (Fig. 4E, blue arrow) via colocalization with the glial marker *repo*.

Finally, we checked whether other insects (e.g., *Drosophila*) also exhibit expression of chemoreceptor genes in nonneuronal antennal cells. We accessed the antennal snRNA-seq data generated as part of the Fly Cell Atlas initiative (*35*) and visualized the expression pattern of all *Gr*, *Ir*, and *Or* genes on the UMAP. In contrast to *H. saltator*, no *Or* was expressed outside ORNs in *Drosophila melanogaster*. However, we identified four *Ir* genes with nonneuronal expression. Specifically, *Ir41a* was expressed in *sv*-positive populations likely representing support cells, and *Ir47a*, *Ir47b*, and *Ir60a* were expressed in different populations of glia as identified by the expression of *repo* (fig. S3B). Similar to the *Or*^+^, *orco*^−^ nonneuronal cells in *H. saltator*, the nonneuronal cells expressing *Ir* genes in *D. melanogaster* did not express any *irco*. Also, leaky regulation appears plausible in at least some of these cases, as *Ir60a* and a closely located gene, *CG3376*, exhibit overlapping expression in some nonneuronal cells (*35*). Thus, *D. melanogaster* also exhibits expression of chemoreceptors in support and glial cells of the antennae, which does not overlap with the expression of their co-receptors. However, the chemoreceptor class expressed is not *Ors* as in *H. saltator*, but *Irs*.

## DISCUSSION

We and others previously found that null mutations in *orco* lead to a wide range of neuronal, physiological, and behavioral defects in ants and honeybees (*15*, *16*, *19*). Here, we further reveal that developing ORNs undergo massive apoptosis in *orco* mutant ants, providing a cellular mechanism underlying their defective neural development.

ORNs start to die at D16 APF in mutant ants, with apoptosis peaking at D19 and D22. Massive apoptosis occurs after the onset of expression of *orco* and *Or* genes (*orco* mRNA peaks at D16, and Orco protein plateaus at D19). Thus, there appears to be a developmental switch around D15 to D19: At this point, Orco abundance increases to reach its peak, and it simultaneously becomes essential for the survival of ORNs (Fig. 2E). As a note, *orco* mutants exhibit a drastically reduced number of glomeruli in the AL (*16*): Only ~20% of glomeruli remain. This can be explained by the lack of axon projection due to ORN cell death, suggesting that axons from most ORNs have not yet projected to glomeruli when massive ORN apoptosis occurs in the mutants (D19 to D22) (Fig. 2E). This suggests that *Or* genes are expressed before axon targeting in *H. saltator*, consistent with the evidence in another ant *O. biroi* (*36*).

Why might Orco be required for the survival of developing ORNs? In *Drosophila*, Orco is required for the trafficking of OR proteins to dendrites (*27*). Disrupted trafficking may lead to an excess of OR protein in the ER of ant ORNs, which in turn causes ER stress and may induce apoptosis. Three lines of evidence, including (i) the death of all tuning *Or*-expressing ORNs, (ii) the survival of ORNs that express *orco,* tuning *Ir* and *irco*, but no tuning *Or*, and (iii) the expression of the ER stress marker phospho-eIF2α at D19 APF in *orco* mutant antennae, are consistent with this hypothesis. However, some *Or*s are expressed in nonneuronal cells that do not express *orco*. This seems inconsistent with apoptosis induction by ER stress, although *Or* expression in nonneurons tends to be lower than their neuronal expression, and the nonneuronal cells might exhibit other properties rendering resistance to ER stress. In *Drosophila*, lack of *orco* triggers neuronal cell death in adults. Cell death occurs early in the maxillary palps (~7 days after eclosion), while in the antennae, loss of neurons occurs around 14 days after eclosion (*18*). This difference might also be explained by differential *Or* expression levels or differential resistance to ER stress, which merits further analysis.

We have identified *Or*-expressing cells that survive in mutant ants. For instance, *HsOr370* is expressed in both neuronal and nonneuronal cells. The latter are likely support cells located near the antennal cuticle that surrounds the dendrites of ORNs, and these cells do not die in the *orco* mutant. There are three types of support cells as identified in *Drosophila*: thecogen (sheath) cells, trichogen (shaft) cells, and tormogen (socket) cells (*37*, *38*). It remains unclear which cell type expresses *HsOr370*. *HsOr202* is coexpressed with *repo*, a glial cell marker. When we reanalyzed the published data from *Drosophila* snRNA-seq (*35*), we found that a few *Ir* but no *Or* genes are expressed in glial and support cells. As these cells are derived from the same developmental lineage as ORNs (*35*, *37*), it is tempting to speculate that certain *Drosophila Ir* and *Harpegnathos Or* genes are turned on in nonneuronal cells under the same regulatory process as in ORNs. Leaky *Or* expression has been observed in mammals, where *Or* genes are coexpressed with neighboring non-*Or* genes in nonneuronal cells (*29*). We found (i) similar spatial expression profiles between *Or* genes and their non-*Or* neighbors and (ii) gradually faded expression of *Or* genes in the *Or* cluster depending on their distance to the non-*Or* gene expressed in these cells, consistent with the notion of leaking expression. However, some *Or* expression cannot be explained by their direct neighboring genes. It is possible that these *Or* genes are regulated by distant enhancers due to high-order chromatin structure.

Mounting evidence also revealed the role of chemosensory receptors expressed in nonneuronal cells. In mammals, nonneuronal ORs play a role in development, reproduction, and immune response (*39*–*41*). In *Drosophila*, certain gustatory receptors—such as *Gr28*—and ionotropic receptors—such as *Ir25a* (*irco*) and *Ir21a* (tuning *Ir*)—are expressed in non-chemosensory neurons and mediate temperature sensing (*42*–*44*). In mosquitoes, *orco* and some *Or*s are expressed in testes and sperm cells and may be involved in sperm chemotaxis (*45*). However, the role of ant *Or*s and *Drosophila Ir*s in glia and support cells remains unclear. As ant *Or* genes expressed in non-ORN cells do not coexpress *orco*, it is possible that these ORs are not functional or their functions are independent of olfaction.

Coexpression of chemosensory receptors is a common phenomenon in *Caenorhabditis elegans*, which has many more *Or* genes than sensory neurons (*46*); however, *Or* expression in insects and vertebrates largely follows the rule of “one neuron, one receptor” (*3*, *5*). Recently, exceptions have been found in mosquitoes and *Drosophila*, where some neurons express multiple *Or* genes. *Orco* is also expressed in neurons expressing *irco*s (*47*–*49*). Consistently, we found a wide range of coexpression between different classes of chemosensory receptors in ants. In contrast to dipteran insects where *Ir25a* is commonly coexpressed with *orco*, the main coexpression pair in ants is *orco*-*Ir8a*. Although the precise function remains unclear, the coexpression might provide evolutionary advantages: For example, an IR complex could modulate neuronal activity by altering membrane resistance (*49*) or allow a limited number of neurons to detect many chemical cues from the same target (*47*).

In summary, our study revealed the temporal windows of SOP cell division, as well as patterns of *orco/Or* gene expression and neuronal survival during ant ORN development. Lack of Orco may lead to ER stress, which in turn causes apoptosis in the developing neurons. Hymenoptera have expanded their *Or* gene repertoire, which possibly resulted in unique developmental events among insect chemosensory systems to support eusociality.

## MATERIALS AND METHODS

### Experimental design and model

Animals for HCR and immunostaining were maintained and collected at the University of Florida Department of Biology. Heterozygous females were obtained by crossing mutant males with WT females. Heterozygous and homozygous mutant females used in this study were collected from the offspring of heterozygous females paired with mutant males. Newly pupated individuals were painted with a single colored dot near the posterior end of the abdomen to mark the day on which they underwent pupation. Pupae were then assigned an age at which to be harvested (10 to 28 days APF). Before harvesting, sex was confirmed by examining the body shape of the pupa. Females could be identified by their long mandibles, large heads, and stout bodies. Males, identified by their long antennae, small heads, and narrow bodies, were removed from the study. To prepare pupal antennae for experiments, the pupal casing was removed by clipping a small hole near the base of the pupa’s abdomen and gently pulling the pupa out of its casing from the posterior end. This prevented any potential damage to the antennae, which were then clipped at the base. Bodies were stored at −80°C and later used for genotyping by Sanger sequencing of the mutation site. Antennae were either used immediately or stored in OCT at −80°C after fixation in paraformaldehyde (PFA). To prepare adult antennae for experiments, callow workers (1 to 2 days after eclosion) were collected from WT colonies. Antennae were clipped at the base and were immediately used for experiments.

WT animals used for snRNA-seq were collected at New York University (NYU) Grossman School of Medicine. Six (library 1) or 26 (libraries 2 and 3) adult females of unknown age were collected from stock colonies. Their antennae were clipped at the base and processed for nuclei extraction immediately. *Orco* mutant animals for snRNA-seq were collected at the University of Florida. Homozygous females reach adulthood, but they generally die early and do not leave any progeny, so the mutation is propagated in the laboratory by selecting for heterozygous reproductive females. To collect homozygous mutants for the experiment, antennae of the freshly eclosed female progeny of heterozygous females were clipped at the base, and both the antennae and the remaining bodies were placed at −80°C for storage. These samples were shipped to NYU Grossman School of Medicine on dry ice. DNA was extracted from the remaining bodies and genotyped by Sanger sequencing of the mutation site. Afterward, the antennae of seven individuals that were found to be homozygous mutants were removed from storage and processed for nuclei extraction.

### Immunostaining of pupal antennae

The protocol for immunostaining, adapted from a previous protocol (*16*), is described here. Pupal antennae were fixed in 4% PFA diluted in 1× phosphate-buffered saline (PBS) with 0.3% Triton X-100 (PBST) for 30 min, washed twice with 0.3% PBST, and underwent overnight incubation in 30% sucrose at 4°C. Sections were taken the following day at a thickness of 15 μm, with focus on the most distal portion of the antennae (F5 to F10). If whole antennae are difficult to section, the scape and F1 to F4 may be removed before sectioning. Tissue was fixed on slides using 4% PFA (as prepared previously) for 30 min at room temperature before two washes with 0.3% PBST. The tissues were then incubated overnight at 4°C in a primary antibody solution (1:400 primary antibody from rabbit). After ensuring that all primary antibody is removed from the slide via two 0.3% PBST washes, tissues were incubated at room temperature for 2 hours in a secondary antibody solution containing Alexa Fluor 555 anti-rabbit secondary antibody (1:400, Thermo Fisher Scientific), Alexa Fluor 488 phalloidin (1:200, Thermo Fisher Scientific), and Hoechst (1:1000, Sigma-Aldrich) before being mounted for microscopy.

### HCR RNA-FISH of adult antennae

Our protocol for HCR RNA-FISH was adapted from several protocols established previously [https://www.molecularinstruments.com/, (*47*, *50*)] and is described here. Antennae were harvested from callow female workers (1 to 2 days after eclosion) and fixed in 4% PFA diluted in 1× PBS with 0.1% Triton X-100 for 30 min, washed twice with 0.1% PBST, and underwent overnight incubation in 30% sucrose at 4°C. Sections were taken at a thickness of 10 μm and fixed on slides using 4% PFA (as prepared previously) for 30 min at room temperature. Tissue was then dehydrated and subsequently rehydrated with a graded series of 5-min MeOH/0.1% PBST washes before incubating in a proteinase K (10 μg/ml) solution for 10 min. Slides then underwent a 10-min incubation in probe hybridization buffer warmed to 37°C. Chambers were made for each slide consisting of a coverslip with double-sided tape lining two parallel edges to raise the coverslip slightly after application to the slide. Each probe set (1.6 pmol) in 100 μl of warmed probe hybridization buffer was added beneath the raised coverslip chamber. The chamber was sealed using rubber cement, and the slides were incubated overnight at 37°C. Probes were removed using a graded series of 15-min washes with probe wash buffer and 5× SSC with 0.1% PBST before incubating at room temperature for 30 min in amplification buffer. For each amplifier set, 6 pmol of hairpin h1 and 6 pmol of hairpin h2 were prepared by heating at 95°C for 90 s and cooling to room temperature in a dark space for at least 30 min. These were quickly added to 100 μl of room temperature amplification buffer. Again, coverslip chambers were created and added to slides, and the amplification buffer mixture was added before the chamber was sealed with rubber cement and the slides were left overnight to incubate at room temperature in a dark space. The amplification buffer mixture was removed via two 30-min washes with 5× SSC and 0.1% PBST before incubation with Hoechst (1:1000) for 15 min. Following two subsequent 30-min washes with 0.1% PBST, slides were mounted for microscopy using Vectashield mounting medium.

### Microscopy and image generation

All tissue samples were imaged using an Olympus IX81-DSU Spinning Disk confocal microscope at the University of Florida Interdisciplinary Center for Biotechnology Research. Z-stacks were acquired with 1 μm between each focal plane for all samples. Images were generated and colorized using Fiji (ImageJ). Immunofluorescence images were quantified by calculating the proportion of stained cell area that was Orco or caspase positive. Given the low variability between replicates (as seen in Fig. 1B), three replicates were typically quantified for each experimental condition. FISH images were quantified by counting the total number of cells in the image and identifying the number of cells with probe colocalization using Fiji’s Cell Counter plugin.

### Bulk RNA-seq

Antennae for bulk RNA-seq were frozen with liquid nitrogen and ground into a fine dry powder using a pestle. All bulk antennal RNA was extracted via a standard protocol using TRIzol Reagent, followed by ethanol precipitation. Deoxyribonuclease treatment was performed in solution, followed by a second round of TRIzol-chloroform and ethanol precipitation to remove the treatment. To generate libraries, we used the NEBNext Ultra II RNA Library Prep Kit for Illumina with an input of 110 ng of RNA and 12 polymerase chain reaction (PCR) cycles. Pooled libraries were sequenced in several rounds using either HiSeq2500 or NovaSeq6000 sequencing systems.

### Nuclei extraction

Nuclei extraction protocol, largely adapted from fly and mosquito protocols (*35*, *47*, *48*), is described below. Dounce homogenizer, pluriStrainers, and tubes were prewetted with the corresponding buffer before adding the sample to prevent the adhesion of nuclei and to minimize sample loss.

1. Prepare a solution containing 1% bovine serum albumin (BSA) and 1 mM RNaseOUT in 1× PBS, pH 7.4 (PBS-BSA).

2. Prepare homogenization buffer following the recipe in table S4.

3. Chill a metal cup on dry ice and chill a pestle by immersing it into liquid nitrogen.

4. If starting with fresh tissue, flash-freeze antennae in liquid nitrogen.

5. Empty freshly frozen antennae or antennae previously stored at −80°C into the cup and grind them while keeping the cup on dry ice.

6. Place the cup on wet ice until thawed.

7. Add 1 ml of homogenization buffer and wash down remaining sample from the pestle and the walls of the cup.

8. Transfer the entire sample into a Dounce homogenizer and release nuclei by 20 strokes of loose, 20 strokes of tight, 20 strokes of tight pestle (avoid foaming with steady consistent motion). Briefly chill on wet ice between the stroking series.

9. Split the suspension into two halves. Do the following for each half: filter the suspension through a 40-μm Flowmi strainer directly into a 20-μm pluriStrainer inserted into a 1.5-ml tube.

10. Centrifuge both tubes for 10 min at 500*g* at 4°C.

11. Without disturbing the pellet (which is not always formed), discard the supernatant.

12. Resuspend each sample in 250 μl of PBS-BSA by pipetting 20 times.

13. Filter each sample three times through a 40-μm Flowmi strainer and finally into a 10-μm pluriStainer inserted into a 1.5-ml tube.

14. Combine the two sample halves.

### Nuclei sorting and snRNA-seq library prep

Nuclear suspensions were stained with Hoechst (5 μg/ml) and processed on the FACSAria II (BD Biosciences) cell sorter. First, single particles were gated in forward scatter (FSC)-A versus FSC-W coordinates, and the resulting population was plotted in Hoechst versus FSC-A coordinates (fig. S5A). Two or three subpopulations with varying and largely overlapping FSC-A signal but highly uniform and distinct levels of Hoechst fluorescence appeared on the plot, consistent with previous reports of nuclei with different ploidy in fly nuclear suspensions (*35*). All such subpopulations were included into the gate used for sorting. Additionally, a fraction of particles had variable but intermediate (higher than unstained control but lower than the nuclear “bands”) levels of Hoechst fluorescence. These particles were considered debris and were not included. Sorted nuclei were collected into a 1.5-ml tube containing 20 μl of PBS-BSA. In total, 43.2 μl (or less, if the volume was insufficient) of the final suspension was used as an input into single-cell RNA-seq library preparation using Chromium Next GEM Single Cell 3′ Kit v3.1 (10X Genomics), which was done following the manufacturer’s instructions. Between 13 and 15 cDNA amplification cycles were performed, and the amount of amplified cDNA used for library prep varied between 64 and 79 ng. Eleven cycles of sample index PCR were performed. Libraries 1 to 3 (WT) were sequenced on a NovaSeq 6000 (Illumina), and the mutant library was sequenced on NextSeq 500 (Illumina). The sequencing configuration was 28–base pair (bp) read 1 + 91-bp read 2 + 8-bp index for library 1 (WT), which was single-indexed, and 28-bp read 1 + 90-bp read 2 + 10-bp index 1 + 10-bp index 2 for libraries 2 to 4 (WT, WT, and mutant), which were double-indexed. Targeted sequencing depth was greater than 20,000 reads per cell.

### Quantification and statistical analysis

#### Receptor gene annotation

Zhou *et al.* (*51*) curated a set of gene annotations for chemoreceptors in *H. saltator*. The genes were classified into *Or*s, *Ir*s, and *Gr*s and given numbers (e.g., *HsOr322*). However, these annotations were generated using an older *H. saltator* genome assembly (GenBank accession GCA_000147195.1) (*52*), which has been since superseded by a more contiguous assembly (GenBank accession GCA_003227715.2) (*53*). Given that Zhou *et al.* annotations have already been used in multiple studies on ant *Or*s (*54*–*57*), we sought to transfer these annotations onto the new assembly and manually curate them while keeping the naming system as consistent as possible. First, we identified potential *Or* and *Gr* genes in the most up-to-date set of *H. saltator* gene annotations generated for the new assembly, HSAL51 [Gene Expression Omnibus (GEO) accession GSE172309] (*58*). To do this, we scanned HSAL51 translations against the hidden Markov model profiles of the PFAM domains 7tm_6 (PF02949) and 7tm_7 (PF08395) (*59*) using HMMER v3.3.2 (http://hmmer.org). Then, we extracted the mRNA sequences of the candidate genes identified in this manner and performed reciprocal BLASTn search between them and the mRNA sequences from Zhou *et al.* using blast_rbh (*60*, *61*). This strategy allowed us to assign IDs to most genes, but a sizable fraction of genes remained unmatched. Also, IR genes from the new assembly were not included in the analysis until this point. Therefore, we performed manual matching and curation of the remaining genes using additional manual reciprocal BLASTn searches and tBLASTn searches against the new assembly. We also made use of the fact that most chemoreceptor genes in *H. saltator* are clustered in the genome, some clusters comprising tens of tandemly arranged genes (*51*). As an example, Zhou *et al.* annotations contain genes *HsOr80*, *HsOr81*, and *HsOr82*, located head to tail next to each other, and HSAL51 annotations contain genes *LOC105191307*, *LOC105191308*, and *LOC105191309* in the same orientation (fig. S5B). Only *LOC105191307* and *LOC105191309* showed up as the best reciprocal BLAST hits of *HsOr80* and *HsOr82*, respectively. *HsOr81*, the gene in the middle, displayed the highest BLAST score against *LOC105191309* = *HsOr82*, but it actually had higher sequence identity to *LOC105191308*. Most likely, *LOC105191308* was not the highest scoring hit because the gene model is truncated, resulting in a shorter alignment and thus a lower score. Nevertheless, we interpreted its higher sequence identity to *HsOr81* and its location relative to its well-matched neighbors as evidence of *LOC105191308* being *HsOr81*. Moreover, we used the chance to manually correct the gene model of *LOC105191308* and make it more consistent with the transcriptomic data generated earlier (*51*) and in this study. Thus, using synteny as a guide and occasionally supplementing it with additional BLAST searches, we identified the absolute majority of Zhou *et al.* genes in the new genome assembly, creating or updating gene models were necessary. There were several cases left when no one-to-one correspondence could be established. One example is shown in fig. S5C: The region of the old assembly containing *HsOr142* and *HsOr143* (scaffold305:48831-53859) appears to be either an erroneous duplication in the old assembly or collapsed with a similar neighboring region in the new one. In either case, these genes are not present as distinct sequences in the new assembly. On the other hand, *HsOr181*, *HsOr182*, *HsOr183*, and *HsOr184* all had identifiable best reciprocal BLAST hits in HSAL51, but the corresponding genomic region in the new assembly contained one extra OR gene. We arbitrarily named it *HsOr182.2*, while the original best hit of *HsOr182* was named *HsOr182.1*. The final set of gene annotations was designated HSAL60 and deposited at GEO. The list of chemoreceptor genes identified in the new genome assembly is provided in table S1 along with the description of what type of evidence was used to assign a Zhou *et al.*–style ID to each gene.

#### Bulk RNA-seq of pupal antennae at different developmental stages

Reads were mapped to the genome, and reads overlapping gene predictions were counted using STAR v2.6.1d (*62*) with the following parameters: --alignIntronMax 7000 --quantMode GeneCounts. Since one of the sequencing batches had longer reads, reads in this batch were trimmed by appropriately specifying the --clip3pNbases parameter of STAR. Gene counts were normalized using DESeq2 v1.34.0 (*63*). To plot the temporal trend of *Or* expression in WT versus mutant, the expression of each *Or* gene was first averaged across replicates and then scaled to its maximum value across genotypes and stages. Next, we calculated the mean and SD of the scaled expression across genes for each genotype and stage. To perform PCA, we first transformed the data using the DESeq2 function vst with default parameters. Then, PCA was done either with *Or* gene set only or with the entire transcriptome. As whole transcriptome–based principal components (PCs) 1 and 2 have separated the samples by developmental stage, we identified the genes driving this separation by examining 750 genes with the largest positive loading on PC1 (presumable early stage–biased genes), 750 genes with the largest negative loading on PC2 (presumable intermediate stage–biased genes), and 750 genes with the largest negative loading on PC1 (presumable late stage–biased genes). To verify the temporal expression pattern of each of these three gene sets, we plotted the mean ± SD of their expression at each stage and for each genotype as described for the *Or* genes above. Finally, we performed Gene Ontology (GO) term enrichment using topGO v2.46.0 (*64*) in conjunction with ViSEAGO v1.8.0 (*65*). In all enrichment analyses, genes with detectable expression, defined as genes with an average sample count of at least 10, were used as the background gene set (*66*). For GO term annotations, previously published Biological Process annotations of *H. saltator* genes (*50*) were intersected with terms present in GO.db v3.14.0 (*67*). topGOdata objects were created with the following parameters: ont = “BP,” nodeSize = 5. GO term enrichment test was performed for “early,” “intermediate,” and “late” genes separately using the following parameters: algorithm = “elim,” statistic = “Fisher.”

#### snRNA-seq preprocessing and clustering

Conversion of raw sequencing data to FASTQ, creation of the transcriptome index, and read counting to generate expression matrix was done in cellranger v7.0.0 with default parameters (*68*). Subsequent analyses were done using scanpy v1.8.2 (*69*). First, cell-wise and gene-wise filtering was applied as follows. Cells with fewer than 750 detected genes, greater than 12,500 Unique Molecular Identifiers (UMIs), or greater than 2.5% mitochondrial reads were removed from the analysis. Then, genes annotated in the National Center for Biotechnology Information (NCBI) file All_Invertebrates.gene_info (https://ftp.ncbi.nih.gov/gene/DATA/GENE_INFO/Invertebrates/All_Invertebrates.gene_info.gz) as “large subunit ribosomal RNA” were removed. First, the different libraries were merged (AnnData.concatenate) and analyzed without performing any batch correction, except what scanpy documentation calls “lightweight batch correction” at the stage of variable gene selection. The combined counts were depth-normalized (CP10k) and log+1 transformed using default parameters. Highly variable gene selection was done with the following parameters: layer = “counts,” batch_key = “orco,” flavor = “seurat_v3,” n_top_genes = 2000, where “orco” is the metadata attribute that encodes whether the library is WT or mutant. PCA was performed, and the optimal number of PCs (*12*) was chosen as the point at which the proportion of variance explained by each PC plateaued. The neighborhood graph and UMAP were computed with default parameters except the number of PCs. Plotting library ID and quality control metrics on the UMAP revealed the following: (i) Even WT libraries prepared from different batches of biological material on different days, i.e. library 1 versus libraries 2 and 3, exhibited pronounced batch effects; (ii) quality metrics, e.g., the number of UMIs, appeared to have a strong effect on cell clustering. Thus, we reanalyzed the data by applying batch correction and increasing the stringency of cell-wise filtering. In addition to the filtering cutoffs applied above, cells with greater than 2750 detected genes, greater than 9500 UMIs, or greater than 1.25% mitochondrial reads were removed. The filtered raw counts were depth-normalized (CP10k) and log+1 transformed using default parameters. Highly variable gene selection was done with the following parameters: layer = “counts,” batch_key = “sample,” flavor = “seurat_v3,” n_top_genes = 2000, where “sample” is the metadata attribute that contains the library ID (1, 2, 3, or 4). Then, scvi-tools v0.16.2 (*70*) was used to set up an scVI model with layer = “counts,” batch_key = “sample,” and then train the model with the following parameters: max_epochs = 800, early_stopping = True, deterministic = True. Obtained latent representation was used to compute the neighborhood graph and UMAP, and Leiden clustering was performed with an arbitrary selected resolution of 5.

#### Cell type annotation

Next, we set out to annotate the cell types. First, cells were broadly split into neurons and nonneurons. Neurons were defined as cells that belonged to clusters that simultaneously expressed *LOC105189534/nSyb*, *LOC105190174/fne*, *LOC105183410/Syt1*, and *LOC105183587/onecut*, while the rest of the cells were classified as nonneuronal cells. Next, given that different ORN types may primarily differ by the expression of only one or several receptor genes and that they may not be represented in our dataset in large numbers, we decided against assigning ORN types to clusters of cells and instead classified each individual cell. To overcome the sparsity of 10X data, we used the following strategy: A Mann-Whitney *U* test (scipy.stats.mannwhitneyu) was performed for each receptor gene to compare its expression in the previously defined neighborhood of the focal cell and in a randomly chosen set of 100 WT nonneuronal cells. A *P* value cutoff of 0.05 was used to label each cell as expressing or not expressing a given gene (fig. S3A). Then, each individual neuron was classified as described in table S5. *LOC105188823/Rh50* and *LOC105188598/nompC* were chosen as markers of ammonia sensory and mechanosensory neurons, respectively, following (*47*, *71*). Finally, cells that belong to clusters expressing *LOC105181500/repo* were classified as glia (*72*), *LOC105191850/Mhc* was used as the marker of muscle cells (*35*), *LOC105181616/grh*-positive cells were classified as epithelium (*35*), and the expression of *LOC105187024/sv* was used to identify neuronal support cells (*35*, *37*).

#### OR phylogeny

To reconstruct the phylogeny of the HSAL60 *Or* genes, we first identified the longest predicted transcript of each gene. Then, we translated them using TransDecoder v5.5.0 (https://github.com/TransDecoder/TransDecoder, accessed 29 December 2022) and only retained a single translation per transcript by passing the --single_best_only flag to TransDecoder.Predict. The translations were aligned using MAFFT v7.508 (*73*) with default parameters. The alignment was visually examined using Jalview v2.11.2.4 (*74*), and sites deemed non-informative were manually removed. Then, phylogenetic tree was built by running RAxML v8.2.12 (*75*) with the following parameters: -f a, -m PROTGAMMAAUTO, -# 100. The best-scoring tree was re-rooted with Orco as an outgroup using FigTree v1.4.4 (http://tree.bio.ed.ac.uk/software/figtree/). The tree in the Newick format is provided as data S1.

#### Bulk RNA-seq of adult antennae

Reads were mapped to the genome, and reads overlapping gene predictions were counted using STAR v2.6.1d (*62*) with the following parameters: --alignIntronMax 7000 --quantMode GeneCounts. The counts were normalized using DESeq2 v1.34.0 (*63*) and overlaid on the OR tree using ggtree v3.2.1 (*76*).

#### Identification of Ors, which retained expression in the orco mutant

We used both the single-nucleus data and the bulk data to identify such genes. Raw single-nucleus counts were depth-normalized (CP10k), and the average expression of each *Or* gene in mutant cells was divided by its average expression in WT cells. The resulting fold change values were log_2_-transformed. Only genes with non-zero expression in both WT and mutant samples were considered. Similarly, fragments per kilobase of transcript per million mapped reads (FPKM) values from the bulk data (see above) were averaged across WT and mutant samples, and log_2_ fold change between mutant and WT was calculated for each OR gene. Plotting log_2_ fold changes in single-nucleus and bulk data revealed a highly significant positive relationship (Pearson *R*^2^ = 0.21, *P* < 10^−13^, Spearman *R*^2^ = 0.17, *P* < 10^−11^) (fig. S4A). To select genes that exhibited the smallest amount of change in the mutant, we drew an arbitrary cutoff for both data sets (fig. S4A). To identify the markers of nonneuronal cells that express *HsOr370*, we performed iterative marker searches using sc.tl.rank_genes_groups with either “rest” or various individual clusters as the reference. The expression specificity was visually assessed by plotting the expression of potential marker genes on the UMAP.

## Supplementary Materials

### This PDF file includes:

Figs. S1 to S5 Legends for tables S1 to S6 Legend for data S1 References
