# Supplementary material for: Orco-dependent survival of odorant receptor neurons in ants
Source: Sci Adv. 2024 Jun 7;10(23):eadk9000. doi: 10.1126/sciadv.adk9000 (PMC11160473; doi:10.1126/sciadv.adk9000)
Supplement: Supplementary file 1 — Figs. S1 to S5 Legends for tables S1 to S6 Legend for data S1 References [file sciadv.adk9000_sm.pdf]

Science Advances
AAAS

Supplementary Materials for

Orco-dependent survival of odorant receptor neurons in ants

Bogdan Sieriebriennikov *et al.*

Corresponding author: Hua Yan, hua.yan@ufl.edu

Sci. Adv. **10**, eadk9000 (2024)
DOI: 10.1126/sciadv.adk9000

The PDF file includes:

Figs. S1 to S5
Legends for tables S1 to S6
Legend for data S1
References

Other Supplementary Material for this manuscript includes the following:

Tables S1 to S6
Data S1

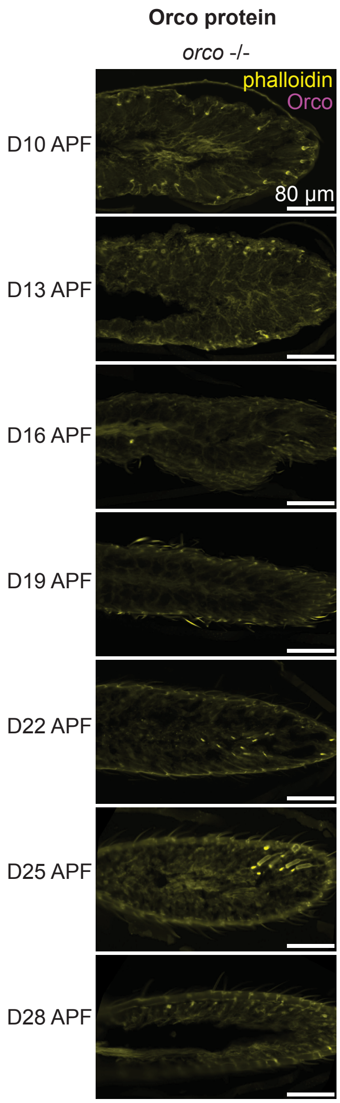

Figure S1. Control stainings in the *orco* mutant.

Representative immunohistochemistry images of sectioned mutant pupal antennae following the staining and age conventions of Fig. 1E. This represents a control for our Orco antibody, as mutant animals should not exhibit positive signal.

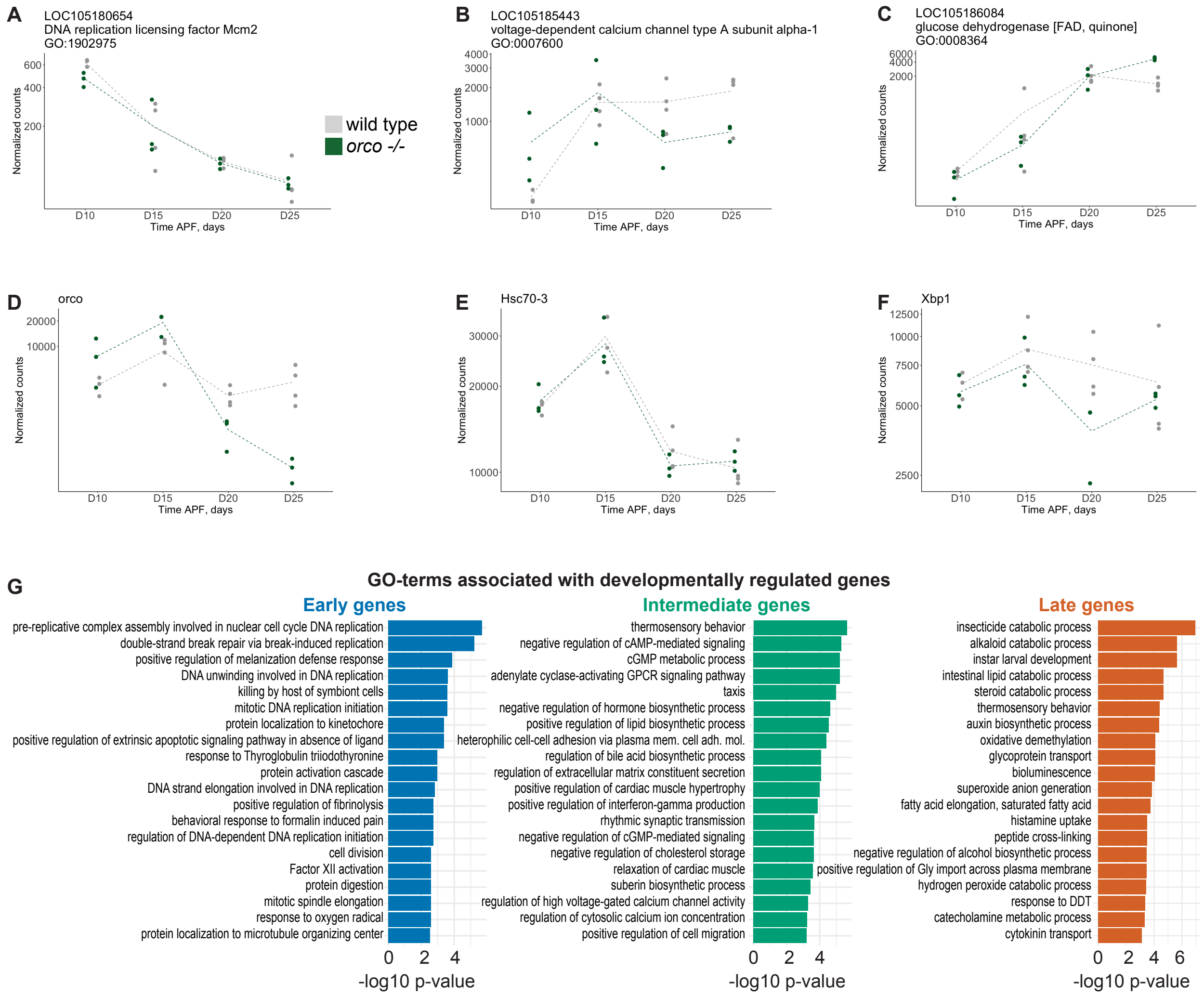

Figure S2. Expression of individual genes in bulk RNA-seq and enriched GO-terms.

(A-C) Expression patterns of selected genes from “early” (A), “intermediate” (B), and “late” (C) gene sets as defined in Fig. 2B. The title of each plot contains gene ID, NCBI annotation, and an associated GO-term significantly enriched in the corresponding gene set. Note that genes shown may be associated with additional GO-terms

(D) Expression pattern of *Orco*.

(E-F) Expression patterns of selected markers of ER stress. The Y axis in panels (A-F) displays DESeq2-normalized gene counts. Each point corresponds to a sample. The dashed lines connect time point means.

(G) Top GO-terms enriched in “early”, “intermediate”, and “late” genes, along with corresponding Fisher’s test p-values.

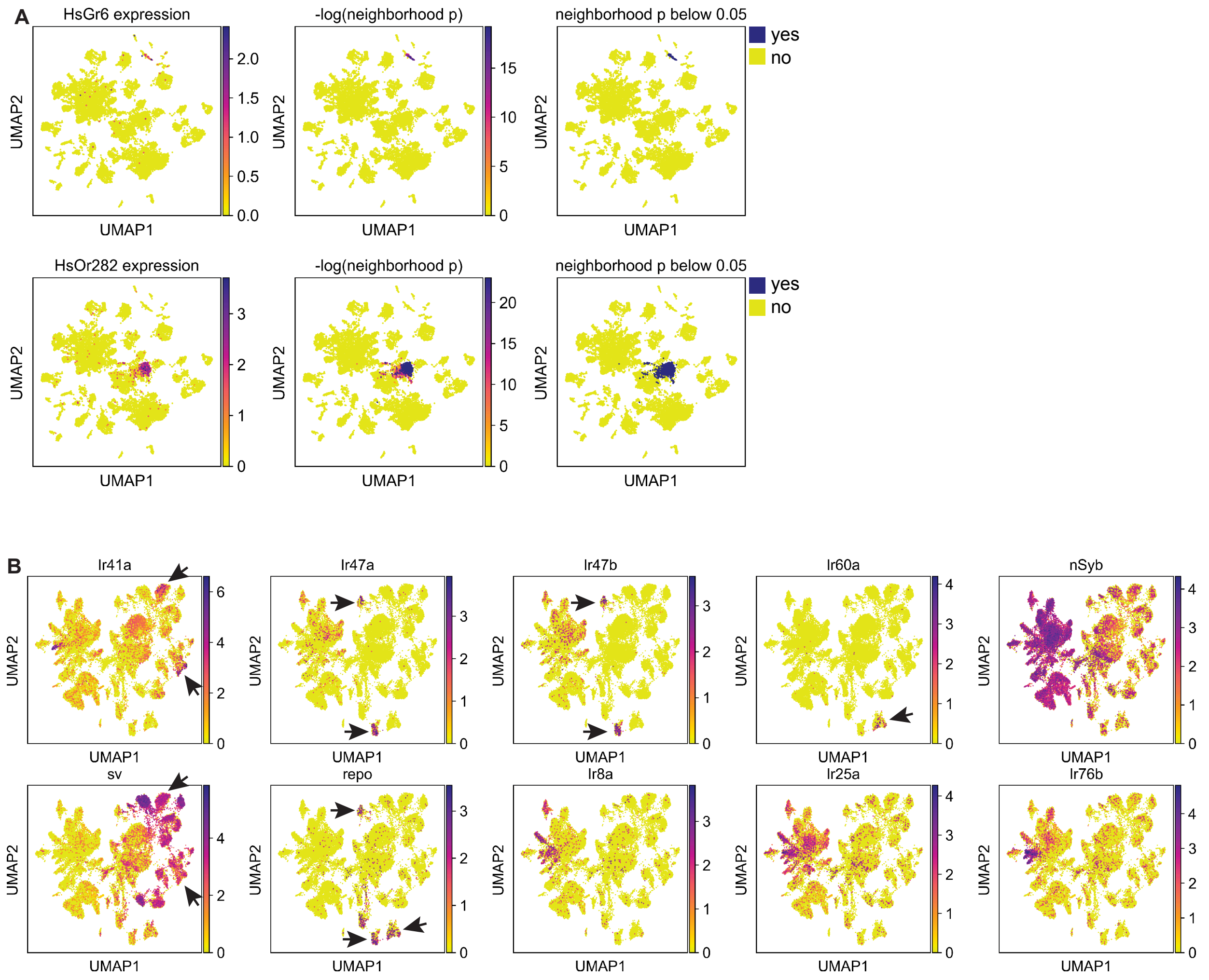

Figure S3. Binarization of gene expression in the *H. saltator* snRNA-seq data and non-neuronal expression of chemoreceptor genes in *D. melanogaster.*

(A) UMAP plots showing the expression of two selected receptor genes. Plots in the left column show depth-normalized and log-transformed counts. Plots in the middle column show the negative logarithm of the p-value obtained from the Mann-Whitney U test between the neighborhood of each cell and a randomly chosen set of 100 wild-type non-neuronal cells (see Methods for details). Plots in the right column show binarized p-values from the middle column (above or below 0.05), which were used to classify cells as expressing or not expressing different receptor classes shown in Fig. 3C.

(B) UMAP plots showing the expression of the four non-neuronally expressed *Ir*s, a neuronal marker *nSyb*, a support cell marker *sv*, a glial cell marker *repo*, and all three *irco*s in *Drosophila*.

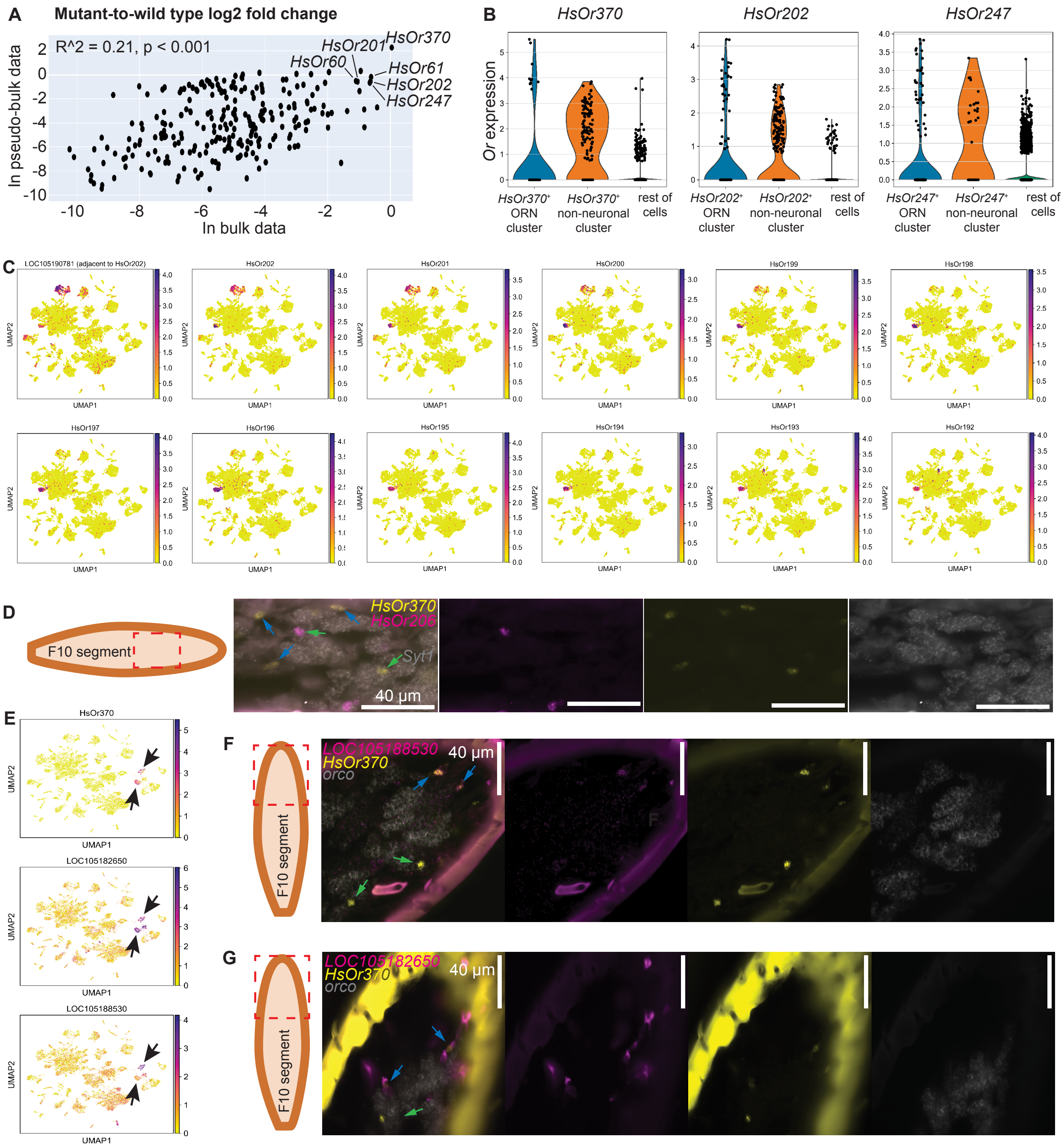

Figure S4. Identification of *Or*s that retain their expression in the mutant and their non-neuronal expression.

(A) Pearson's correlation between the level of expression retention (expressed as a log2 fold change in *Or* expression between mutant and WT) in bulk data and in pseudo-bulk data.

(B) Expression level of selected *Or*s from fig. 4 in ORNs and non-neurons.

(C) UMAP plots showing the expression of *Or*s *HsOr202-HsOr193* and their upstream non-*Or* neighbor.

(D) Representative HCR RNA-FISH images of sectioned WT adult antennae illustrating the relationships between an *Or* with exclusively neuronal expression (*HsOr206*, magenta), an *Or* with additional non-neuronal expression (*HsOr370*, yellow), and a neuronal marker (*Syt1*, grey). *HsOr206* always co-localizes with the neuronal marker (left green arrow). *HsOr370* may co-localize with the neuronal marker (right green arrow) or without (blue arrows). The checked red box in the diagram illustrates the region of the flagellum (as illustrated in Fig. 1A) imaged.

(E) UMAP plots showing the expression of *HsOr370* and of two genes marking the *HsOr370*-positive support cells. Arrows point at the support cell clusters that express *HsOr370*.

(F-G) Representative HCR RNA-FISH images of sectioned WT adult antennae. Here, we show the relationship between *HsOr370* (yellow), identified support cell markers (magenta) and a neuronal marker (orco, grey). Green arrows show cases of *HsOr370* expression in neuronal cells. Blue arrows demonstrate cases where *HsOr370* co-localizes (D) or “nests” (E) with support cell markers, but not the neuronal marker. The checked red box in the diagram illustrates the region of the flagellum (as illustrated in Fig. 1A) imaged.

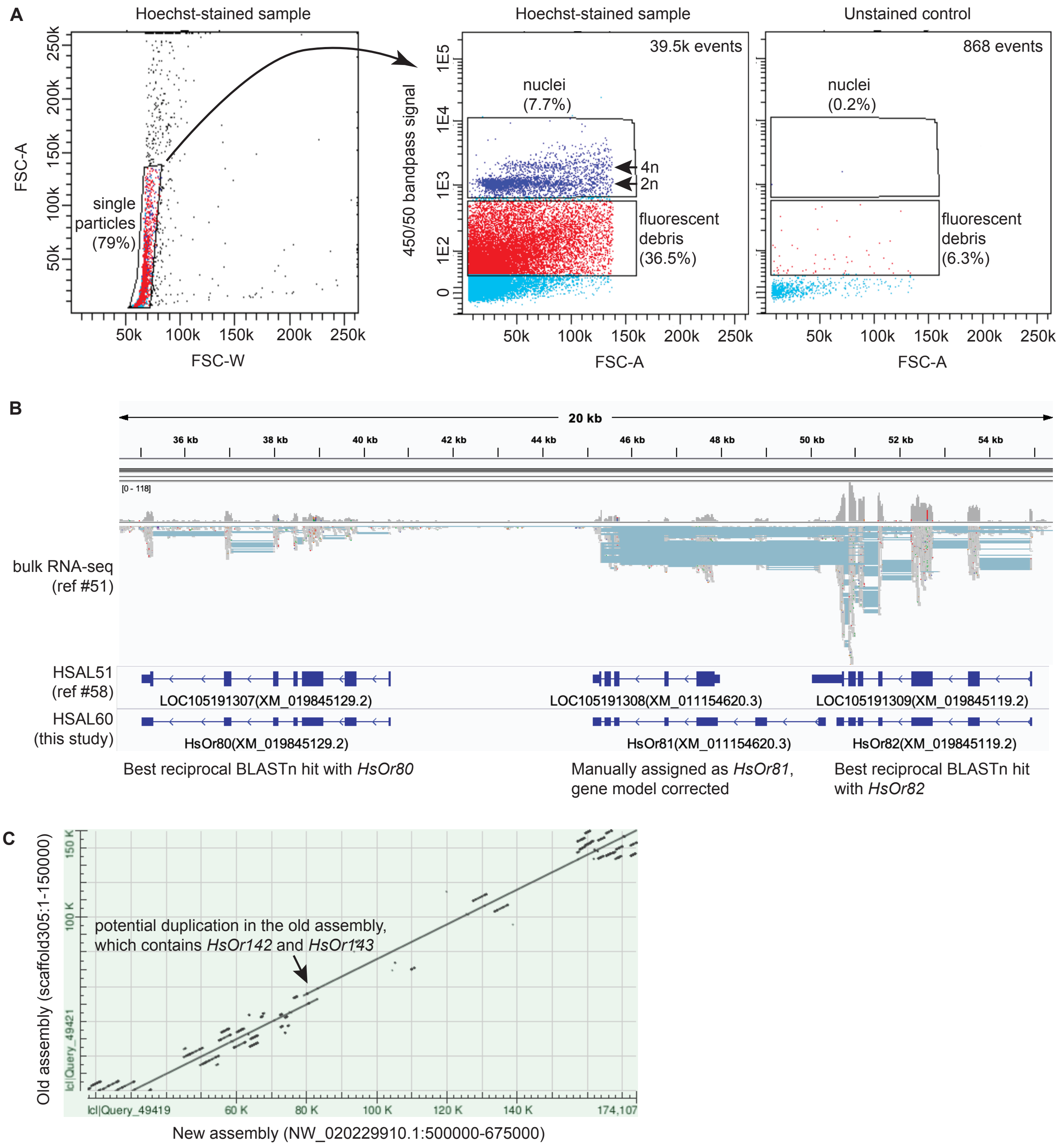

Figure S5. Additional experimental details.

(A) FACS gating. FSC = forward scatter, A = area, W = width, 2n = putative diploid nuclei, 4n = putative tetraploid nuclei. The "nuclei" gate was used for sorting.

(B) Genome browser snapshot showing the locus that contains *HsOr80, HsOr81,* and *HsOr82* along with aligned bulk RNA-seq data, HSAL51 and HSAL60 gene annotations, and the

description of evidence used to transfer gene IDs from Zhou et al. (51) onto the HSAL60 annotations.

(C) Dot plot output of BLAST between the region that contains *HsOr142* and *HsOr143* in the old genome assembly and the homologous region in the new assembly.

Table S1. List of *Gr*, *Ir*, and *Or* genes in the HSAL60 annotation set.

Table S2. List of GO-terms enriched in “early”, “intermediate”, and “late” gene sets.

Table S3. List of *Or* loci, the description of their expression pattern in mutant snRNA-seq data, and neighboring non-*Or* genes which could potentially drive non-neuronal expression of *Or*s.

Table S4. Recipe of the homogenization buffer.

Table S5. Classification strategy for neuronal cell types. “+” stands for expression (neighborhood p-value < 0.05) and “**-**“ stands for the lack of expression.

Table S6. A table of key resources.

Data S1. Phylogenetic tree of *Or* genes in the Newick format.

REFERENCES AND NOTES

1. J. M. Smith, E. Szathmáry, *The Major Transitions in Evolution* (Oxford Univ. Press, 1998).

2. E. O. Wilson, B. Hölldobler, Eusociality: Origin and consequences. *Proc. Natl. Acad. Sci. U.S.A.* **102**, 13367–13371 (2005).

3. T. Komiyama, L. Luo, Development of wiring specificity in the olfactory system. *Curr. Opin. Neurobiol.* **16**, 67–73 (2006).

4. H. M. Robertson, Molecular evolution of the major arthropod chemoreceptor gene families. *Annu. Rev. Entomol.* **64**, 227–242 (2019).

5. H. Yan, S. Jafari, G. Pask, X. Zhou, D. Reinberg, C. Desplan, Evolution, developmental expression and function of odorant receptors in insects. *J. Exp. Biol.* **223**, jeb208215 (2020).

6. T. Imai, H. Sakano, Odorant receptor-mediated signaling in the mouse. *Curr. Opin. Neurobiol.* **18**, 251–260 (2008).

7. S. Rengarajan, E. A. Hallem, Olfactory circuits and behaviors of nematodes. *Curr. Opin. Neurobiol.* **41**, 136–148 (2016).

8. R. Benton, On the ORigin of smell: Odorant receptors in insects. *Cell Mol. Life Sci.* **63**, 1579–1585 (2006).

9. J. A. Butterwick, J. del Mármol, K. H. Kim, M. A. Kahlson, J. A. Rogow, T. Walz, V. Ruta, Cryo-EM structure of the insect olfactory receptor Orco. *Nature* **560**, 447–452 (2018).

10. K. Sato, M. Pellegrino, T. Nakagawa, T. Nakagawa, L. B. Vosshall, K. Touhara, Insect olfactory receptors are heteromeric ligand-gated ion channels. *Nature* **452**, 1002–1006 (2008).

11. K. Touhara, L. B. Vosshall, Sensing odorants and pheromones with chemosensory receptors. *Annu. Rev. Physiol.* **71**, 307–332 (2009).

12. D. Wicher, R. Schäfer, R. Bauernfeind, M. C. Stensmyr, R. Heller, S. H. Heinemann, B. S. Hansson, Drosophila odorant receptors are both ligand-gated and cyclic-nucleotide-activated cation channels. *Nature* **452**, 1007–1011 (2008).

13. P. Brand, H. M. Robertson, W. Lin, R. Pothula, W. E. Klingeman, J. L. Jurat-Fuentes, B. R. Johnson, The origin of the odorant receptor gene family in insects. *eLife* **7**, e38340 (2018).

14. C. Opachaloemphan, H. Yan, A. Leibholz, C. Desplan, D. Reinberg, Recent advances in behavioral (Epi)genetics in eusocial insects. *Annu. Rev. Genet.* **52**, 489–510 (2018).

15. W. Trible, L. Olivos-Cisneros, S. K. McKenzie, J. Saragosti, N. Chang, B. J. Matthews, P. R. Oxley, D. J. C. Kronauer, *orco* mutagenesis causes loss of antennal lobe glomeruli and impaired social behavior in ants. *Cell* **170**, 727–735.e10 (2017).

16. H. Yan, C. Opachaloemphan, G. Mancini, H. Yang, M. Gallitto, J. Mlejnek, A. Leibholz, K. Haight, M. Ghaninia, L. Huo, M. Perry, J. Slone, X. Zhou, M. Traficante, C. A. Penick, K. Dolezal, K. Gokhale, K. Stevens, I. Fetter-Pruneda, R. Bonasio, L. J. Zwiebel, S. L. Berger, J. Liebig, D. Reinberg, C. Desplan, An engineered *orco* mutation produces aberrant social behavior and defective neural development in ants. *Cell* **170**, 736–747.e9 (2017).

17. A. Chiang, P. Rashi, M. Ramaswami, K. Vijayraghavan, V. Rodrigues, Neuronal activity and Wnt signaling act through Gsk3-β to regulate axonal integrity in mature Drosophila olfactory sensory neurons. *Development* **136**, 1273–1282 (2009).

18. D. Task, C. J. Potter, Rapid degeneration of Drosophila olfactory neurons in Orco mutant maxillary palps. *MicroPubl. Biol.* (2021).

19. Z. Chen, I. M. Traniello, S. Rana, A. C. Cash-Ahmed, A. L. Sankey, C. Yang, G. E. Robinson, Neurodevelopmental and transcriptomic effects of CRISPR/Cas9-induced somatic *orco* mutation in honey bees. *J. Neurogenet.*, **3**, 320–332 (2021).

20. C. R. Yu, J. Power, G. Barnea, S. O'Donnell, H. E. V. Brown, J. Osborne, R. Axel, J. A. Gogos, Spontaneous neural activity is required for the establishment and maintenance of the olfactory sensory map. *Neuron* **42**, 553–566 (2004).

21. H. J. Shayya, J. K. Kahiapo, R. Duffié, K. S. Lehmann, L. Bashkirova, K. Monahan, R. P. Dalton, J. Gao, S. Jiao, I. Schieren, L. Belluscio, S. Lomvardas, ER stress transforms random olfactory receptor choice into axon targeting precision. *Cell* **185**, 3896–3912.e22 (2022).

22. C. A. Penick, S. S. Prager, J. Liebig, Juvenile hormone induces queen development in late-stage larvae of the ant Harpegnathos saltator. *J. Insect Physiol.* **58**, 1643–1649 (2012).

23. K. Endo, T. Aoki, Y. Yoda, K.-i. Kimura, C. Hama, Notch signal organizes the Drosophila olfactory circuitry by diversifying the sensory neuronal lineages. *Nat. Neurosci.* **10**, 153–160 (2007).

24. A. Sen, D. Kuruvilla, L. Pinto, A. Sarin, V. Rodrigues, Programmed cell death and context dependent activation of the EGF pathway regulate gliogenesis in the Drosophila olfactory system. *Mech. Dev.* **121**, 65–78 (2004).

25. P. C. Chai, S. Cruchet, L. Wigger, R. Benton, Sensory neuron lineage mapping and manipulation in the Drosophila olfactory system. *Nat. Commun.* **10**, 643 (2019).

26. S. Barish, P. C. Volkan, Mechanisms of olfactory receptor neuron specification in Drosophila. *Wiley Interdiscip. Rev. Dev. Biol.* **4**, 609–621 (2015).

27. M. C. Larsson, A. I. Domingos, W. D. Jones, M. E. Chiappe, H. Amrein, L. B. Vosshall, Or83b encodes a broadly expressed odorant receptor essential for Drosophila olfaction. *Neuron* **43**, 703–714 (2004).

28. L. Wang, H. D. Ryoo, Y. Qi, H. Jasper, PERK limits drosophila lifespan by promoting intestinal stem cell proliferation in response to ER stress. *PLOS Genet.* **11**, e1005220 (2015).

29. E. Feldmesser, T. Olender, M. Khen, I. Yanai, R. Ophir, D. Lancet, Widespread ectopic expression of olfactory receptor genes. *BMC Genomics* **7**, 121 (2006).

30. C. Flegel, S. Manteniotis, S. Osthold, H. Hatt, G. Gisselmann, Expression profile of ectopic olfactory receptors determined by deep sequencing. *PLOS ONE* **8**, e55368 (2013).

31. E. A. Feingold, L. A. Penny, A. W. Nienhuis, B. G. Forget, An olfactory receptor gene is located in the extended human β-globin gene cluster and is expressed in erythroid cells. *Genomics* **61**, 15–23 (1999).

32. P. T. Spellman, G. M. Rubin, Evidence for large domains of similarly expressed genes in the Drosophila genome. *J. Biol.* **1**, 5 (2002).

33. P. Quintero-Cadena, P. W. Sternberg, Enhancer sharing promotes neighborhoods of transcriptional regulation across eukaryotes. *G3 (Bethesda)* **6**, 4167–4174 (2016).

34. S. Prelic, V. P. Mahadevan, V. Venkateswaran, S. Lavista-Llanos, B. S. Hansson, D. Wicher, Functional interaction between Drosophila olfactory sensory neurons and their support cells. *Front. Cell. Neurosci.* **15**, 789086 (2022).

35. H. Li, J. Janssens, M. De Waegeneer, S. S. Kolluru, K. Davie, V. Gardeux, W. Saelens, F. P. A. David, M. Brbic, K. Spanier, J. Leskovec, C. N. McLaughlin, Q. Xie, R. C. Jones, K. Brueckner, J. Shim, S. G. Tattikota, F. Schnorrer, K. Rust, T. G. Nystul, Z. Carvalho-Santos, C. Ribeiro, S. Pal, S. Mahadevaraju, T. M. Przytycka, A. M. Allen, S. F. Goodwin, C. W. Berry, M. T. Fuller, H. White-Cooper, E. L. Matunis, S. DiNardo, A. Galenza, L. E. O'Brien, J. A. T. Dow, F. C. A. C. S. Sign, H. Jasper, B. Oliver, N. Perrimon, B. Deplancke, S. R. Quake, L. Luo, S. Aerts, D. Agarwal, Y. Ahmed-Braimah, M. Arbeitman, M. M. Ariss, J. Augsburger, K. Ayush, C. C. Baker, T. Banisch, K. Birker, R. Bodmer, B. Bolival, S. E. Brantley, J. A. Brill, N. C. Brown, N. A. Buehner, X. T. Cai, R. Cardoso-Figueiredo, F. Casares, A. Chang, T. R. Clandinin, S. Crasta, C. Desplan,

A. M. Detweiler, D. B. Dhakan, E. Dona, S. Engert, S. Floc'hlay, N. George, A. J. Gonzalez-Segarra, A. K. Groves, S. Gumbin, Y. Guo, D. E. Harris, Y. Heifetz, S. L. Holtz, F. Horns, B. Hudry, R. J. Hung, Y. N. Jan, J. S. Jaszczak, G. Jefferis, J. Karkanias, T. L. Karr, N. S. Katheder, J. Kezos, A. A. Kim, S. K. Kim, L. Kockel, N. Konstantinides, T. B. Kornberg, H. M. Krause, A. T. Labott, M. Laturney, R. Lehmann, S. Leinwand, J. Li, J. S. S. Li, K. Li, L. Li, T. Li, M. Litovchenko, H. H. Liu, Y. Liu, T. C. Lu, J. Manning, A. Mase, M. Matera-Vatnick, N. R. Matias, C. E. McDonough-Goldstein, A. McGeever, A. D. McLachlan, P. Moreno-Roman, N. Neff, M. Neville, S. Ngo, T. Nielsen, C. E. O'Brien, D. Osumi-Sutherland, M. N. Ozel, I. Papatheodorou, M. Petkovic, C. Pilgrim, A. O. Pisco, C. Reisenman, E. N. Sanders, G. Dos Santos, K. Scott, A. Sherlekar, P. Shiu, D. Sims, R. V. Sit, M. Slaidina, H. E. Smith, G. Sterne, Y. H. Su, D. Sutton, M. Tamayo, M. Tan, I. Tastekin, C. Treiber, D. Vacek, G. Vogler, S. Waddell, W. Wang, R. I. Wilson, M. F. Wolfner, Y. E. Wong, A. Xie, J. Xu, S. Yamamoto, J. Yan, Z. Yao, K. Yoda, R. Zhu, R. P. Zinzen, Fly Cell Atlas: A single-nucleus transcriptomic atlas of the adult fruit fly. *Science* **375**, eabk2432 (2022).

36. A. R. Ryba, S. K. McKenzie, L. Olivos-Cisneros, E. J. Clowney, P. M. Pires, D. J. C. Kronauer, Comparative development of the Ant chemosensory system. *Curr. Biol.* **30**, 3223–3230.e4 (2020).

37. M. Scalzotto, R. Ng, S. Cruchet, M. Saina, J. Armida., C. Su, R. Benton, Pheromone sensing in Drosophila requires support cell-expressed Osiris 8. *BMC Biol.* **20**, 230 (2022).

38. H. R. Schmidt, R. Benton, Molecular mechanisms of olfactory detection in insects: Beyond receptors. *Open Biol.* **10**, 200252 (2020).

39. N. Kang, J. Koo, Olfactory receptors in non-chemosensory tissues. *BMB Rep.* **45**, 612–622 (2012).

40. C. Schneider, C. E. O'Leary, R. M. Locksley, Regulation of immune responses by tuft cells. *Nat. Rev. Immunol.* **19**, 584–593 (2019).

41. A. D. Workman, J. N. Palmer, N. D. Adappa, N. A. Cohen, The role of bitter and sweet taste receptors in upper airway immunity. *Curr. Allergy Asthma Rep.* **15**, 72 (2015).

42. C. Chen, E. Buhl, M. Xu, V. Croset, J. S. Rees, K. S. Lilley, R. Benton, J. J. L. Hodge, R. Stanewsky, Drosophila ionotropic receptor 25a mediates circadian clock resetting by temperature. *Nature* **527**, 516–520 (2015).

43. L. Ni, M. Klein, K. V. Svec, G. Budelli, E. C. Chang, A. J. Ferrer, R. Benton, A. D. Samuel, P. A. Garrity, The ionotropic receptors IR21a and IR25a mediate cool sensing in Drosophila. *eLife* **5**, e13254 (2016).

44. L. Ni, P. Bronk, E. C. Chang, A. M. Lowell, J. O. Flam, V. C. Panzano, D. L. Theobald, L. C. Griffith, P. A. Garrity, A gustatory receptor paralogue controls rapid warmth avoidance in Drosophila. *Nature* **500**, 580–584 (2013).

45. R. J. Pitts, C. Liu, X. Zhou, J. C. Malpartida, L. J. Zwiebel, Odorant receptor-mediated sperm activation in disease vector mosquitoes. *Proc. Natl. Acad. Sci. U.S.A.* **111**, 2566–2571 (2014).

46. B. Vidal, U. Aghayeva, H. Sun, C. Wang, L. Glenwinkel, E. A. Bayer, O. Hobert, An atlas of Caenorhabditis elegans chemoreceptor expression. *PLOS Biol.* **16**, e2004218 (2018).

47. M. Herre, O. V. Goldman, T. C. Lu, G. Caballero-Vidal, Y. Qi, Z. N. Gilbert, Z. Gong, T. Morita, S. Rahiel, M. Ghaninia, R. Ignell, B. J. Matthews, H. Li, L. B. Vosshall, M. A. Younger, Non-canonical odor coding in the mosquito. *Cell* **185**, 3104–3123.e28 (2022).

48. C. N. McLaughlin, M. Brbic, Q. Xie, T. Li, F. Horns, S. S. Kolluru, J. M. Kebschull, D. Vacek, A. Xie, J. Li, R. C. Jones, J. Leskovec, S. R. Quake, L. Luo, H. Li, Single-cell

transcriptomes of developing and adult olfactory receptor neurons in Drosophila. *eLife* **10**, e63856 (2021).

49. D. Task, C. C. Lin, A. Vulpe, A. Afify, S. Ballou, M. Brbic, P. Schlegel, J. Raji, G. Jefferis, H. Li, K. Menuz, C. J. Potter, Chemoreceptor co-expression in Drosophila melanogaster olfactory neurons. *eLife* **11**, e72599 (2022).

50. H. Yan, C. Opachaloemphan, F. Carmona-Aldana, G. Mancini, J. Mlejnek, N. Descostes, B. Sieriebriennikov, A. Leibholz, X. Zhou, L. Ding, M. Traficante, C. Desplan, D. Reinberg, Insulin signaling in the long-lived reproductive caste of ants. *Science* **377**, 1092–1099 (2022).

51. X. Zhou, J. D. Slone, A. Rokas, S. L. Berger, J. Liebig, A. Ray, D. Reinberg, L. J. Zwiebel, Phylogenetic and transcriptomic analysis of chemosensory receptors in a pair of divergent ant species reveals sex-specific signatures of odor coding. *PLOS Genet.* **8**, e1002930 (2012).

52. R. Bonasio, G. Zhang, C. Ye, N. S. Mutti, X. Fang, N. Qin, G. Donahue, P. Yang, Q. Li, C. Li, P. Zhang, Z. Huang, S. L. Berger, D. Reinberg, J. Wang, J. Liebig, Genomic comparison of the ants *Camponotus floridanus* and *Harpegnathos saltator*. *Science* **329**, 1068–1071 (2010).

53. E. J. Shields, L. Sheng, A. K. Weiner, B. A. Garcia, R. Bonasio, High-quality genome assemblies reveal long non-coding RNAs expressed in ant brains. *Cell Rep.* **23**, 3078–3090 (2018).

54. P. Engsontia, U. Sangket, H. M. Robertson, C. Satasook, Diversification of the ant odorant receptor gene family and positive selection on candidate cuticular hydrocarbon receptors. *BMC Res. Notes* **8**, 380 (2015).

55. G. M. Pask, J. D. Slone, J. G. Millar, P. Das, J. A. Moreira, X. Zhou, J. Bello, S. L. Berger, R. Bonasio, C. Desplan, D. Reinberg, J. Liebig, L. J. Zwiebel, A. Ray, Specialized

odorant receptors in social insects that detect cuticular hydrocarbon cues and candidate pheromones. *Nat. Commun.* **8**, 297 (2017).

56. R. Saad, A. B. Cohanim, M. Kosloff, E. Privman, Neofunctionalization in ligand binding sites of ant olfactory receptors. *GBE* **10**, 2490–2500 (2018).

57. J. D. Slone, G. M. Pask, S. T. Ferguson, J. G. Millar, S. L. Berger, D. Reinberg, J. Liebig, A. Ray, L. J. Zwiebel, Functional characterization of odorant receptors in the ponerine ant, Harpegnathos saltator. *Proc. Natl. Acad. Sci. U.S.A.* **114**, 8586–8591 (2017).

58. E. J. Shields, M. Sorida, L. Sheng, B. Sieriebriennikov, L. Ding, R. Bonasio, Genome annotation with long RNA reads reveals new patterns of gene expression and improves single-cell analyses in an ant brain. *BMC Biol.* **19**, 254 (2021).

59. J. Mistry, S. Chuguransky, L. Williams, M. Qureshi, G. A. Salazar, E. L. L. Sonnhammer, S. C. E. Tosatto, L. Paladin, S. Raj, L. J. Richardson, R. D. Finn, A. Bateman, Pfam: The protein families database in 2021. *Nucleic Acids Res.* **49**, D412–D419 (2021).

60. C. Camacho, G. Coulouris, V. Avagyan, N. Ma, J. Papadopoulos, K. Bealer, T. L. Madden, BLAST+: Architecture and applications. *BMC Bioinformatics* **10**, 421 (2009).

61. P. J. A. Cock, J. M. Chilton, B. Grüning, J. E. Johnson, N. Soranzo, NCBI BLAST+ integrated into Galaxy. *GigaScience* **4**, 39 (2015).

62. A. Dobin, C. A. Davis, F. Schlesinger, J. Drenkow, C. Zaleski, S. Jha, P. Batut, M. Chaisson, T. R. Gingeras, STAR: Ultrafast universal RNA-seq aligner. *Bioinformatics* **29**, 15–21 (2013).

63. M. I. Love, W. Huber, S. Anders, Moderated estimation of fold change and dispersion for RNA-seq data with DESeq2. *Genome Biol.* **15**, 550 (2014).

64. A. Alexa, J. Rahnenfuhrer. topGO: Enrichment analysis for gene ontology. R package version 2.54.0 (2023).

65. A. Brionne, A. Juanchich, C. Hennequet-Antier, ViSEAGO: A Bioconductor package for clustering biological functions using Gene Ontology and semantic similarity. *BioData Min.* **12**, 16 (2019).

66. K. Wijesooriya, S. A. Jadaan, K. L. Perera, T. Kaur, M. Ziemann, Urgent need for consistent standards in functional enrichment analysis. *PLOS Comput. Biol.* **18**, e1009935 (2022).

67. M. Carlson. GO.db: A set of annotation maps describing the entire Gene Ontology. R package version 3.8.2 (2019).

68. G. X. Zheng, J. M. Terry, P. Belgrader, P. Ryvkin, Z. W. Bent, R. Wilson, S. B. Ziraldo, T. D. Wheeler, G. P. McDermott, J. Zhu, M. T. Gregory, J. Shuga, L. Montesclaros, J. G. Underwood, D. A. Masquelier, S. Y. Nishimura, M. Schnall-Levin, P. W. Wyatt, C. M. Hindson, R. Bharadwaj, A. Wong, K. D. Ness, L. W. Beppu, H. J. Deeg, C. McFarland, K. R. Loeb, W. J. Valente, N. G. Ericson, E. A. Stevens, J. P. Radich, T. S. Mikkelsen, B. J. Hindson, J. H. Bielas, Massively parallel digital transcriptional profiling of single cells. *Nat. Commun.* **8**, 14049 (2017).

69. F. A. Wolf, P. Angerer, F. J. Theis, SCANPY: Large-scale single-cell gene expression data analysis. *Genome Biol.* **19**, 15 (2018).

70. A. Gayoso, R. Lopez, G. Xing, P. Boyeau, V. V. Pour Amiri, J. Hong, K. Wu, M. Jayasuriya, E. Mehlman, M. Langevin, Y. Liu, J. Samaran, G. Misrachi, A. Nazaret, O. Clivio, C. Xu, T. Ashuach, M. Gabitto, M. Lotfollahi, V. Svensson, E. da Veiga Beltrame, V. Kleshchevnikov, C. Talavera-Lopez, L. Pachter, F. J. Theis, A. Streets, M. I. Jordan, J. Regier, N. Yosef, A Python library for probabilistic analysis of single-cell omics data. *Nat. Biotechnol.* **40**, 163–166 (2022).

71. A. Vulpe, K. Menuz, Ir76b is a co-receptor for amine responses in Drosophila olfactory neurons. *Front. Cell. Neurosci.* **15**, 759238 (2021).

72. K. Davie, J. Janssens, D. Koldere, M. De Waegeneer, U. Pech, L. Kreft, S. Aibar, S. Makhzami, V. Christiaens, C. Bravo Gonzalez-Blas, S. Poovathingal, G. Hulselmans, K. I. Spanier, T. Moerman, B. Vanspauwen, S. Geurs, T. Voet, J. Lammertyn, B. Thienpont, S. Liu, N. Konstantinides, M. Fiers, P. Verstreken, S. Aerts, A single-cell transcriptome atlas of the aging Drosophila brain. *Cell* **174**, 982–998.e20 (2018).

73. K. Katoh, D. M. Standley, MAFFT multiple sequence alignment software version 7: Improvements in performance and usability. *Mol. Biol. Evol.* **30**, 772–780 (2013).

74. A. M. Waterhouse, J. B. Procter, D. M. A. Martin, M. Clamp, G. J. Barton, Jalview Version 2—A multiple sequence alignment editor and analysis workbench. *Bioinformatics* **25**, 1189–1191 (2009).

75. A. Stamatakis, RAxML version 8: A tool for phylogenetic analysis and post-analysis of large phylogenies. *Bioinformatics* **30**, 1312–1313 (2014).

76. G. Yu, Using ggtree to visualize data on tree-like structures. *Curr. Protoc. Bioinformatics* **69**, e96 (2020).

77. J. Schindelin, I. Arganda-Carreras, E. Frise, V. Kaynig, M. Longair, T. Pietzsch, S. Preibisch, C. Rueden, S. Saalfeld, B. Schmid, J. Y. Tinevez, D. J. White, V. Hartenstein, K. Eliceiri, P. Tomancak, A. Cardona, Fiji: An open-source platform for biological-image analysis. *Nat. Methods* **9**, 676–682 (2012).
